# RNAi Screen Reveals Potentially Novel Roles of Cytokines in Myoblast Differentiation

**DOI:** 10.1371/journal.pone.0068068

**Published:** 2013-07-02

**Authors:** Yejing Ge, Rachel J. Waldemer, Ramakrishna Nalluri, Paul D. Nuzzi, Jie Chen

**Affiliations:** Department of Cell and Developmental Biology, University of Illinois at Urbana-Champaign, Urbana, Illinois, United States of America; Muséum National d'Histoire Naturelle, France

## Abstract

Cytokines are cell-secreted signaling molecules that modulate various cellular functions, with the best-characterized roles in immune responses. The expression of numerous cytokines in skeletal muscle tissues and muscle cells has been reported, but their function in skeletal myogenesis, the formation of skeletal muscle, has been largely underexplored. To systematically examine the potential roles of cytokines in skeletal myogenesis, we undertook an RNAi screen of 134 mouse cytokine genes for their involvement in the differentiation of C2C12 myoblasts. Our results have uncovered 29 cytokines as strong candidates for novel myogenic regulators, potentially conferring positive and negative regulation at distinct stages of myogenesis. These candidates represent a diverse collection of cytokine families, including interleukins, TNF-related factors, and chemokines. Our findings suggest the fundamental importance of cytokines in the cell-autonomous regulation of myoblast differentiation, and may facilitate future identification of novel therapeutic targets for improving muscle regeneration and growth in health and diseases.

## Introduction

During embryonic skeletal myogenesis, cells in somites are guided by various environmental cues to undergo myogenic commitment, and pass along the myogenic pathway by terminal differentiation and fusion to form multinucleated myofibers [1], [2]. In adult skeletal muscles, injury or other remodeling cues induce satellite cell activation and proliferation, followed by differentiation to form new myofibers or repair existing ones [3]. Skeletal myogenesis *in vivo* can be largely recapitulated by differentiation of cultured myoblasts, which follows a series of ordered steps including cell cycle withdrawal, myogenic protein expression, cell elongation, migration, and fusion to form myotubes [4].

Cytokines are broadly defined as cell-secreted signaling proteins that modulate cellular functions. The earliest defined cytokines were those secreted by immune cells to modulate immune responses, such as interleukins [5], [6]. Other major families of cytokines include the TNFα family [7] and the TGFβ family [8]. Chemokines are a family of structurally related cytokines that regulate chemotaxis by signaling through the G protein coupled receptor family of chemokine receptors [9], and are best known for their functions in immune cells. The expression and function of various cytokines in skeletal muscle have also been reported. For instance, myostatin, a TGFβ family member, is expressed almost exclusively in skeletal muscle and negatively regulates muscle mass [10]. On the other hand, the expression of follistatin, which antagonizes myostatin and promotes myocyte fusion [11], [12], is not restricted to muscle. The prototypic immunoregulatory cytokine interleukin-4 (IL-4) has been found to be expressed in skeletal myocytes and play a key role in myoblast recruitment and late-stage fusion to allow growth of myotubes/myofibers [13]. Interleukin-6 (IL-6) is also expressed in muscle [14], [15], and it promotes myogenic differentiation [14], [16] as well as satellite cell proliferation during muscle growth [15]. Some cytokines that reportedly modulate myogenic differentiation have not been shown to be expressed in muscle cells and may act through endocrine or paracrine mechanisms, the bone morphogenic proteins (BMPs) being an example [17], [18].

Injury-induced muscle regeneration is accompanied by immune cell infiltration and inflammatory responses. Many cytokines have been found to be expressed in regenerating muscles (e.g., [19]), but the source of these cytokines can be infiltrating immune cells or muscle cells, or both. Recent proteomic analyses of the secretome of the myogenic C2C12 cells have revealed tens of cytokines and growth factors that are expressed during differentiation in a regulated manner [20], [21]. Another study has identified numerous chemokine mRNAs expressed by differentiating mouse primary myocytes in culture [22], which may be involved in regulating cell migration during myogenesis. It is possible that other processes of myogenic differentiation may also be regulated by various families of cytokines. To systematically examine the potential roles of cytokines in skeletal myogenesis, we took an RNAi approach to screen a large portion of cytokine genes in the mouse genome for their involvement in the differentiation of C2C12 myoblasts. A wide range of cytokines has been identified from this screen as candidates for positive and negative regulators of myogenic differentiation. Based on the knockdown phenotypes, these candidates are divided into four groups. Selected cytokines representing each group have been further confirmed for their roles in myogenic differentiation.

## Materials and Methods

### Reagents

Anti-MHC (MF20) was obtained from the Developmental Studies Hybridoma Bank developed under the auspices of the NICHD, National Institutes of Health and maintained by The University of Iowa, Department of Biological Sciences. Anti-mouse IgG-FITC was from Jackson ImmunoResearch Laboratories, Inc. All shRNA constructs (in the pLKO lentiviral vector) in the form of bacterial glycerol stocks were purchased from Sigma-Aldrich (MISSION® TRC). All other reagents were also from Sigma-Aldrich.

### Cell Culture

C2C12 myoblasts were maintained in DME containing 1 g/L glucose with 10% fetal bovine serum at 37°C with 7.5% CO_2_. To induce differentiation, cells were plated on tissue culture plates coated with 0.2% gelatin and grown to 75–100% confluence before switching to differentiation medium (DME containing 2% horse serum). The cells were replenished with fresh differentiation medium daily for 3 days.

### Lentivirus-mediated RNAi Screen and other shRNA Constructs

Lentivirus packaging was performed as previously described [23], scaled down for the 96-well format. A panel of 134 cytokine genes was selected and for each gene, 2–5 shRNA constructs were used (Table S1). A non-targeting shRNA (Addgene plasmid 1864; [24]) was included as a negative control. For the primary screen, C2C12 myoblasts seeded in 96-well plates were transduced with individual lentiviruses and selected in 3 µg/mL puromycin for 2 days, followed by 3-day differentiation. Each experiment included the control shRNA virus at several titers of viral transduction, resulting in several different cell densities at the time of differentiation. Comparisons were made to the control of similar cell density (nuclei number). Each knockdown was repeated as least 3 times. The secondary screen was performed with C2C12 cells seeded in 12-well plates, and the shRNAs of primary hits were examined in several groups. The control shRNA was included in each group from viral packaging, infection to differentiation for side-by-side comparison. Myocytes at the end of differentiation were fixed and immuno-stained for MHC and DAPI. Myotube formation was quantified by differentiation index, fusion index, and myonuclei number per myotube (see below for detailed description).

### Immunofluorescence Microscopy and Quantitative Analysis of Myocytes

Differentiated C2C12 cells were fixed and stained for MHC and DAPI as previously described [25]. The stained cells were examined with a Leica DMI 4000B fluorescence microscope, and the fluorescent images were captured using a RETIGA EXi camera, and analyzed with Q-capture Pro51 software (Q-Imaging™). The differentiation index (% of nuclei in MHC-positive myocytes), fusion index (% of nuclei in MHC-positive myotubes with at least 2 nuclei), and myonuclei number per myotube were calculated. Each data point was generated from quantifying all cells in 5 randomly chosen microscopic fields, totaling 1000–2500 nuclei.

### RT-PCR

C2C12 cells were lysed in Trizol (Invitrogen), and total RNA was isolated following the manufacturer’s protocol. cDNA was synthesized from 1 µg RNA using qScript cDNA synthesis kit (Quanta Biosciences) according to the manufacturer’s protocol, followed by PCR using gene-specific primers for limited cycles (15–25). β-actin was used as a loading control. The sequences of primers used in this study are shown in Table S2. The PCR bands were quantified by densitometry, and normalized to β-actin control.

### Statistical Analysis

All data are presented as mean ± SD (n≥3). Whenever necessary, statistical significance of the data comparison was analyzed by performing one-sample or paired *t* test. The specific types of tests and the p-values, when applicable, are indicated in the figure legends.

## Results and Discussion

### An RNAi Screen of Cytokines for Novel Regulators of Myoblast Differentiation

To search for cytokines potentially involved in myoblast differentiation in a cell-autonomous manner, we carried out an RNAi-based functional screen in mouse C2C12 myoblasts. In determining the coverage of this screen, we did not limit the gene set to those reported to express in skeletal myocytes or muscles. We reasoned that such expression profiling was incomplete at the time of our screen and, in addition, some *bona fide* myogenic cytokines might be expressed at ultra-low levels in C2C12 cells but nevertheless functional in those cells, IL-4 being an example (our unpublished observations). Instead, the screen was performed with the TRC collection of lentivirus-delivered shRNAs targeting mouse cytokine genes that were not yet reported to have a clear cell-autonomous myogenic function at the time that we initiated the screen. The pLKO vector-based TRC lentivirus system had been used extensively in our previous studies with C2C12 cells, and no toxicity had been found with infection by the control virus expressing a non-targeting hairpin sequence (e.g., [26]).

**Figure 1:**
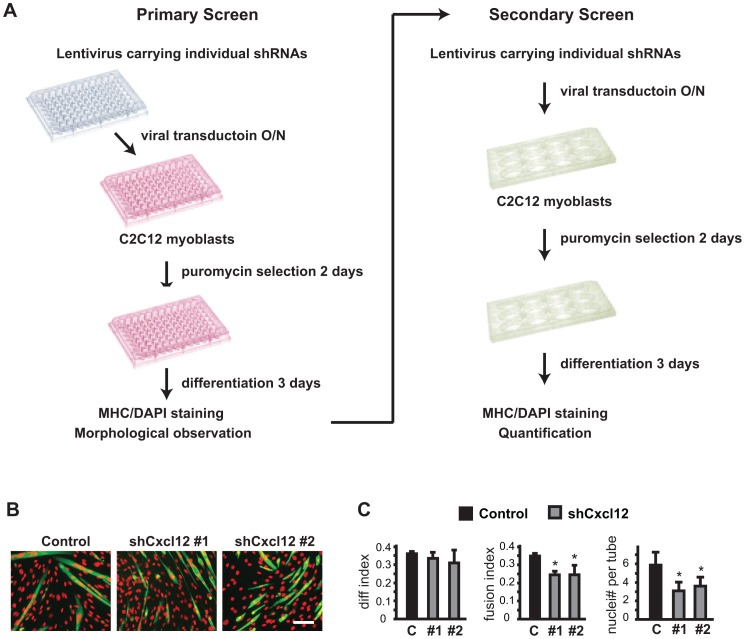
RNAi screen procedure and control. (**A**) A flow chart of the screening process. For the primary screen, lentiviruses expressing shRNAs against 134 genes, packaged in 96-well plates, were used to transduce C2C12 myoblasts seeded in 96-well plates. After 2 days of puromycin selection, the cells were induced to differentiate for 3 days, at the end of which they were fixed and stained for MHC and DAPI. shRNAs that induced morphological changes detectable by visual inspection were subjected to secondary screen with C2C12 cells seeded in 12-well plates, following the procedure described above. Quantification of myotube formation was then performed. (**B**) As a positive control, Cxcl12 was included in the screen and recovered as a positive hit. Shown are results of the secondary screen with two independent shRNAs. Cells were stained for MHC and DAPI, pseudo-colored green and red, respectively. A non-targeting shRNA served as a negative control. (**C**) Myotube formation in B was quantified for differentiation index, fusion index and average nuclei number per myotube (see Material and Methods for definition). Data shown are mean ± SD (n = 3). Paired t test was performed to compare data to control. *P<0.05. Scale bar: 100 µm.

A total of 134 genes was included in the primary screen, each targeted by 2 to 5 distinct shRNA constructs (Table S1), some of which were commercially validated for efficient knockdown. C2C12 myoblasts were infected with lentiviruses expressing shRNAs in a 96-well format, selected with puromycin (for the lentiviral vector), and subsequently induced by serum withdrawal to undergo myogenic differentiation (Fig. 1A). At the end of 3-day differentiation, cells were immuno-stained with the MF-20 antibody, which recognizes all isoforms of sarcomeric myosin heavy chain (MHC) expressed in differentiating myocytes [27], and visually inspected under the microscope for changes in myotube size and number. The primary screen was repeated 3 times, and genes with at least 2 shRNAs consistently eliciting a visible change in myotubes were considered primary hits. The primary hits were then subjected to secondary screen (Fig. 1A). C2C12 cells were seeded in 12-well plates and infected by the shRNA-expressing lentiviruses, followed by puromycin selection and then differentiation for 3 days. Upon MHC and DAPI staining, the myocytes were quantified for three parameters: differentiation index defined as percentage of nuclei in MHC-positive cells, fusion index defined as percentage of nuclei in cells containing 2 or more nuclei (myotubes), and average myonuclei number per myotube as a measurement of myotube size.

The chemokine Cxcl12 (also named SDF-1) was included in the screen as a positive control, since it is expressed by myoblasts and muscle tissues, and regulates myoblast migration and myocyte fusion through its receptor CXCR4 [22], [28], [29], [30]. Cxcl12 was indeed recovered as a positive hit. Cxcl12 knockdown by two independent shRNAs resulted in smaller myotubes as indicated by decreased fusion index and average myonuclei number, without affecting the differentiation index (Fig. 1B&C). This result recapitulates the observation by Griffin et al. in mouse primary myoblasts [22], in full agreement with the reported role of Cxcl12-CXCR4 in regulating myocyte migration and fusion in both primary myoblast [22] and C2C12 cultures [28]. Of note, Odemis et al. [31] reported that recombinant SDF-1 inhibits C2C12 differentiation, which is at odds with our and others’ observations. Although clonal variation of C2C12 could be one explanation for this discrepancy, it should also be pointed out that the results of Odemis et al. came from adding recombinant SDF-1 to the cultures [31] whereas knockdown of SDF-1 was performed by Griffin et al. [22] and in our study. It is possible that SDF-1/Cxcl12 has multiple functions in regulating distinct steps of myogenic differentiation, and different experimental approaches may reveal different functions. Taken together, our observation with Cxcl12 knockdown suggested that the experimental system was sufficiently robust for the screen.

### A Variety of Cytokines are Candidate Regulators of Myoblast Differentiation

**Figure 2:**
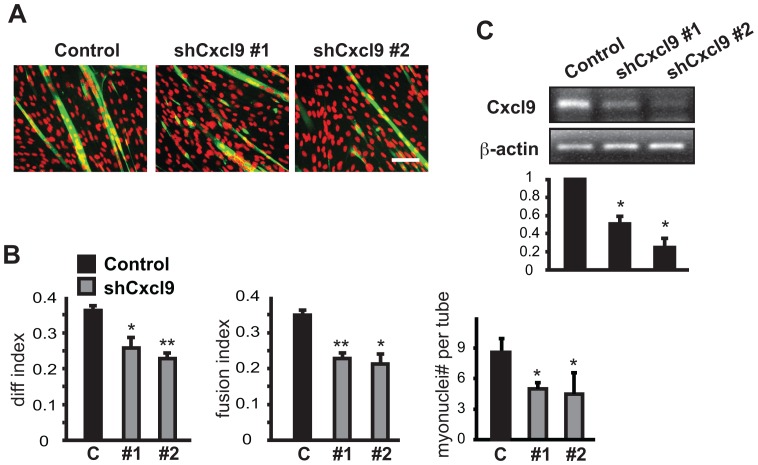
Knockdown of Cxcl9 impairs overall myoblast differentiation. C2C12 myoblasts were transduced overnight with lentiviruses expressing shRNAs for Cxcl9, selected by puromycin for 2 days, and differentiated for 3 days. (**A**) At the end of differentiation, the cells were fixed and immuno-stained for MHC (green), and DAPI stain (red) identified nuclei. Scale bar: 100 µm. (**B**) Myotube formation in A was quantified for differentiation index, fusion index and average nuclei number per myotube. (**C**) Before differentiation, total RNA was isolated from transduced and selected cells, and subjected to RT-PCR. Data shown are mean ± SD (n = 3). Paired (for B) or one-sample (for C) t tests were performed to compare data to control. *P<0.05. **P<0.01.

**Figure 3:**
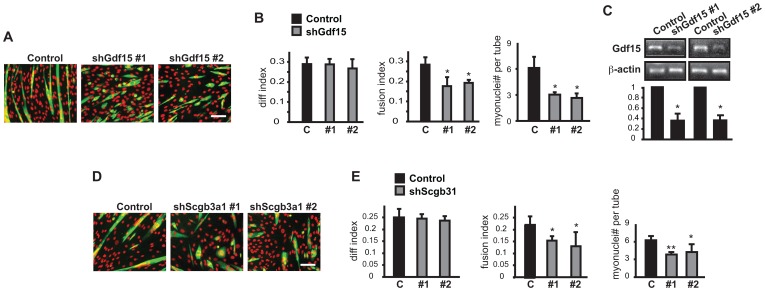
Knockdown of Gdf15 or Scgb3a1 impairs myoblast fusion. C2C12 myoblasts were transduced overnight with lentiviruses expressing shRNAs for Gdf15 (A-C) or Scgb3a1 (D-E) as described in Fig. 2 legend. (**A**) MHC (green) and DAPI (red) staining of Gdf15 knockdown cells at the end of 3-day differentiation. (**B**) Quantification of myotube formation shown in A. (**C**) RT-PCR results for Gdf15 mRNA. (**D**) MHC (green) and DAPI (red) staining of Scgb3a1 knockdown cells at the end of 3-day differentiation. (**E**) Quantification of myotube formation shown in D. Data shown are mean ± SD (n = 3). One sample (C) or paired (B & E) t tests were performed to compare data to control. *P<0.05. **P<0.01. Scale bars: 100 µm.

**Figure 4:**
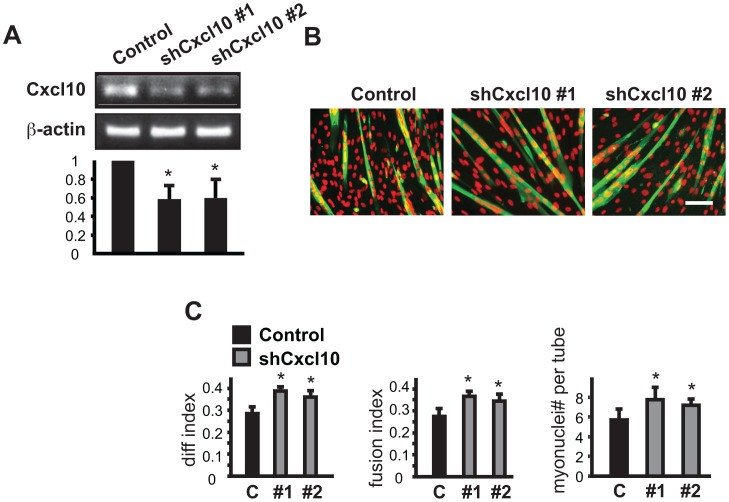
Knockdown of Cxcl10 enhances myoblast differentiation. C2C12 myoblasts were transduced overnight with lentiviruses expressing shRNAs for Cxcl10 as described in Fig. 2 legend. (**A**) RT-PCR results for Cxcl12 mRNA. (**B**) MHC (green) and DAPI (red) staining of Cxcl10 knockdown cells at the end of 3-day differentiation. Scale bar: 100 µm. (**C**) Quantification of myotube formation shown in B. Data shown are mean ± SD (n = 3). One sample (A) or paired (C) t tests were performed to compare data to control. *P<0.05.

**Figure 5:**
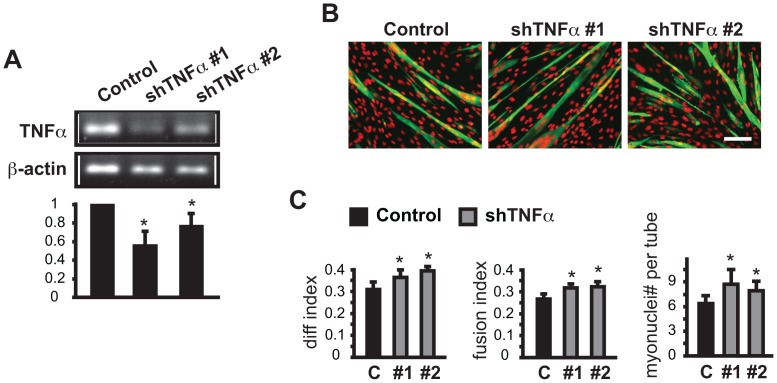
Knockdown of TNFα enhances myoblast differentiation. C2C12 myoblasts were transduced overnight with lentiviruses expressing shRNAs for TNFα as described in Fig. 2 legend. (**A**) RT-PCR results for TNFα mRNA. (**B**) MHC (green) and DAPI (red) staining of TNFα knockdown cells at the end of 3-day differentiation. Scale bar: 100 µm. (**C**) Quantification of myotube formation shown in B. Data shown are mean ± SD (n = 3). One sample (A) or paired (C) t tests were performed to compare data to control. *P<0.05.

**Figure 6:**
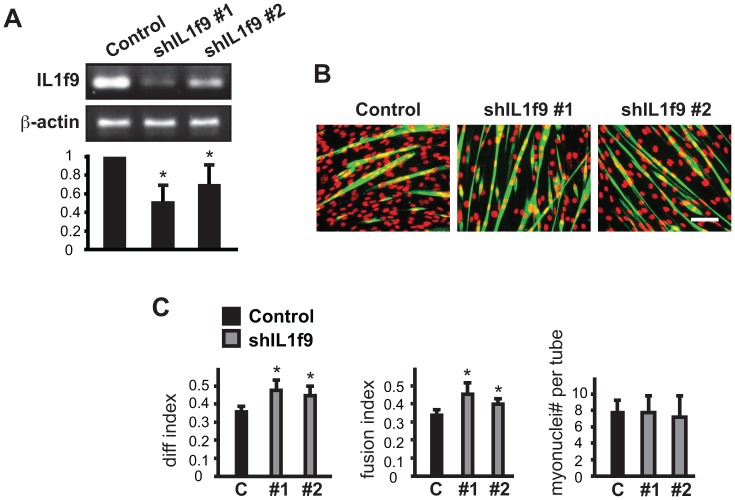
Knockdown of IL1f9 enhances myoblast differentiation without increasing myotube size. C2C12 myoblasts were transduced overnight with lentiviruses expressing shRNAs for IL1f9 as described in Fig. 2 legends. (**A**) RT-PCR results for IL1f9 mRNA. (**B**) MHC (green) and DAPI (red) staining of IL1f9 knockdown cells at the end of 3-day differentiation. Scale bar: 100 µm. (**C**) Quantification of myotube formation shown in B. Data shown are mean ± SD (n = 3). One sample (A) or paired (C) t tests were performed to compare data to control. *P<0.05.

With 2 independent shRNAs for the same gene eliciting consistent phenotype as the criterion, our secondary screen led to the identification of 29 genes (22% of genes screened) as potential regulators of myoblast differentiation (Table 1). The results of myotube quantification for the knockdown of these genes are shown in Table S3, with additional data shown in Figs. 2, 3, 4, 5, 6. These genes represent a diverse collection of cytokines, including interleukins, TNF-related factors, chemokines, and other families. Interestingly, this candidate list consists of a higher number of potentially negative regulators (23) than positive ones (6) (Table 1). Although this may reflect the secretion of a large number of differentiation suppressors by myoblasts to maintain undifferentiated state and homeostasis, it is equally possible that our assay was biased toward revealing an enhanced differentiation phenotype due to sub-optimal differentiation conditions associated with the 96-well format screen.

**Table 1:** RNAi screening identifies potential regulators of myoblast differentiation.

Positive		Negative			
I	II	III		IV	
Ccl8	***Gdf15***	Ccl1	Gdf3	Ccl9	IL18
***Cxcl9***	***Scgb3a1***	Cmtm5	IL5	Ccl17	IL21
Flt3L		Cmtm6	IL17b	Cmtm2a	***IL1f9***
Tnfsf14		Ctf2	IL17c	Ebi3	ILtifb
		Cxcl2	Scg2		
		***Cxcl10***	***Tnfα***		
		Cxcl14	Tnfsf10		
		FasL			

RNAi-based functional screening of 134 mouse cytokine genes revealed 29 candidate regulators of myoblast differentiation, of which 6 are potentially positive regulators, and 23 negative regulators. They are further divided into 4 classes based on knockdown phenotypes. Class I: decrease in all myotube parameters. Class II: decrease in fusion index and average myonuclei number, with unchanged differentiation index. Class III: increase in all myotube parameters. Class IV: increase in differentiation and fusion indexes, with unchanged average myonuclei number. Those highlighted (bold, italic) are examples with data shown in Figs. 2, 3, 4, 5, 6. Data for all the genes listed here are shown in Table S3.

Despite the much smaller size of the positive regulator list, different classes of cytokines are found in each of the two lists, including TNF superfamily members, two major subclasses of chemokines (Ccl and Cxcl families), and others. However, the six interleukins identified are all potential negative regulators, in contrast to the reported positive functions of IL-4 [13] and IL-6 [14], [16] in myogenesis. This may not be surprising given the diverse functions of interleukins in other cellular contexts.

Pavlath and colleagues recently reported the mRNA expression of numerous cytokines/chemokines, their receptors, and related signaling molecules in myoblasts and/or differentiating myocytes [22]. Of the 51 cytokines/chemokines reported to be expressed, 45 are among the 134 genes covered by our RNAi screen, and 12 of them are in our positive hit list (Table 1) in addition to Cxcl12. Henningsen et al. identified 59 cytokines and growth factors secreted by differentiating C2C12 cells in a regulated manner [20], of which 14 were included in our screen and 2 emerged as positive hits (Ccl8 and Gdf15) other than Cxcl12. Because we had limited numbers of shRNAs per gene and adhered to the two-shRNA-per-gene criterion for scoring phenotypic changes, it is possible that we had missed some potential regulators among those shown to be expressed in myocytes.

Based on the phenotypes defined by the 3 parameters – differentiation index, fusion index, and average myonuclei number per myotube, we have further divided the 29 genes into 4 categories – class I-IV (Table 1). The different groups of cytokines likely impinge on various processes of differentiation via distinct mechanisms. Below we describe validation of representative cytokines from each group.

### Class I: Cytokines Required for Initiation of Differentiation

Of the candidates for positive regulators, 4 cytokines (Ccl8, Cxcl9, Flt3L, and Tnfsf14) appeared to regulate an early stage of differentiation because their knockdown led to a decrease in differentiation index, as well as fusion index and myotube size (Table 1 and data not shown). As a representative of this group, the results of Cxcl9 knockdown are shown in Fig. 2A&B. Furthermore, Cxcl9 expression in C2C12 cells, and its knockdown by two independent shRNAs, were confirmed at the mRNA level by RT-PCR (Fig. 2C). Cxcl9 is one of three interferon-induced ligands for the inflammatory chemokine receptor CXCR3, a key regulator of inflammation and a major player in autoimmune diseases [32], [33]. But a function of Cxcl9 in muscle cells has yet to be reported. The other cytokines in this group, Ccl8, Flt3L and Tnfsf14, are also known as regulators of immune responses, and none has been reported to have a function in myogenesis. Although these 4 cytokines elicit a similar phenotype when knocked down, they signal through distinct families of receptors – the Ccl8 and Cxcl9 receptors are GPCRs, the Flt3L receptor is a receptor tyrosine kinase, and the Tnfsf14 receptor belongs to the TNFR superfamily of trimeric receptors. Future characterization of the myogenic signaling pathways activated by these cytokines will likely be informative.

### Class II: Cytokines Regulating Myocyte Fusion

The knockdown of Gdf15 and Scgb3a1 resulted in a distinct phenotype – reduced fusion index and myotube size with unchanged differentiation index, suggesting that, like Cxcl12, these two cytokines may regulate myocyte fusion. Myotube morphology and quantification of the indexes for Gdf15 knockdown by two independent shRNAs are shown in Fig. 3A&B. The expression and RNAi depletion of Gdf15 were confirmed by RT-PCR (Fig. 3C). shRNAs of Scgb3a1 yielded very similar results (Fig. 3D&E). However, Scgb3a1 mRNA was not detected by RT-PCR in C2C12 cells at any stage of differentiation, thus, the knockdown efficiency was not yet confirmed. Nevertheless, the consistent phenotype resulted from two independent shRNAs is likely an on-target effect. It is not impossible that a gene expressed at a level below detection has a key function. IL-4 is such an example. We confirmed the reported function of IL-4 as a fusion factor [13] in C2C12 cells by RNAi, but did not detect IL-4 mRNA in these cells even with commercially validated PCR primers (data not shown).

Gdf15 belongs to the TGFβ superfamily, and has been associated with a range of biological processes, most notably erythropoiesis [34]. Scgb3a1, also known as HIN-1 (high in normal-1), is found to be down-regulated at its gene expression level by hypermethylation in many human cancers [35], [36]. The biochemical mechanism of Scgb3a1 signaling remains elusive.

It is noteworthy that both Gdf15 and Scgb3a1 knockdown resulted in stubby, ball-like myotubes, a morphology resembling that of Brag2-knockdown myotubes reported by Pajcini et al., termed “bragball” [37]. Brag2 is a guanine nucleotide exchange factor, and it controls ARF6 activation and paxillin localization necessary for myoblast elongation and proper fusion [37]. It will be interesting for future studies to examine possible connections between Gdf15 and Scgb3a1 signaling and the Brag2-ARF6-paxillin pathway.

### Class III & IV: Cytokines Inhibiting Differentiation

The majority of candidates identified fell into the group of potential negative regulators. As a representative of class III candidates (Table 1), Cxcl10 knockdown by two independent shRNAs was confirmed (Fig. 4A), and the enhanced myotube formation was evident from myotube morphology (Fig. 4B) and from an increase in all 3 parameters – differentiation index, fusion index, and average size of myotubes (Fig. 4C). Interestingly, Cxcl10 shares the same receptor with Cxcl9– CXCR3 [32], [33]. Our observations suggest that the two ligands may have opposite roles in myogenic differentiation (neither reported before). This may not be too surprising considering that the inter-relationship among the three CXCR3 ligands – Cxcl9, Cxcl10, and Cxcl11– is complex in immune responses, and that redundancy, synergism, and antagonism are all possible [32]. Further investigation of these ligands and their receptor in myogenesis should prove interesting.

The knockdown of another cytokine in class III, TNFα, had very similar effects on myotube formation (Fig. 5). TNFα, as a major proinflammatory cytokine, is secreted by immune cells at sites of muscle injury and found to suppress myoblast differentiation [38], [39]. However, it has also been reported that mechanical stimulation leads to release of TNFα by myoblasts, which is necessary for myogenic differentiation [40]. It was not clear whether the two reported opposing functions of TNFα in myogenic differentiation could be attributed to the different sources of TNFα– in one case from the infiltrating immune cells and the other from muscle cells. Our results for the first time provide evidence that muscle cell-produced TNFα also inhibits differentiation. Apparently, the distinct biological contexts – serum withdrawal versus mechanical stimulation – determine the specific cell-autonomous function of TNFα.

The cytokines in class IV (Table 1) are also candidates of negative regulators. However, they are distinct from those in class III in that their knockdown led to increased differentiation and fusion indexes without a change in average myonuclei number in myotubes. Thus, the higher fusion index was manifested in increased myotube number rather than size. The data for IL1f9 knockdown are shown as an example (Fig. 6).

**Figure 7:**
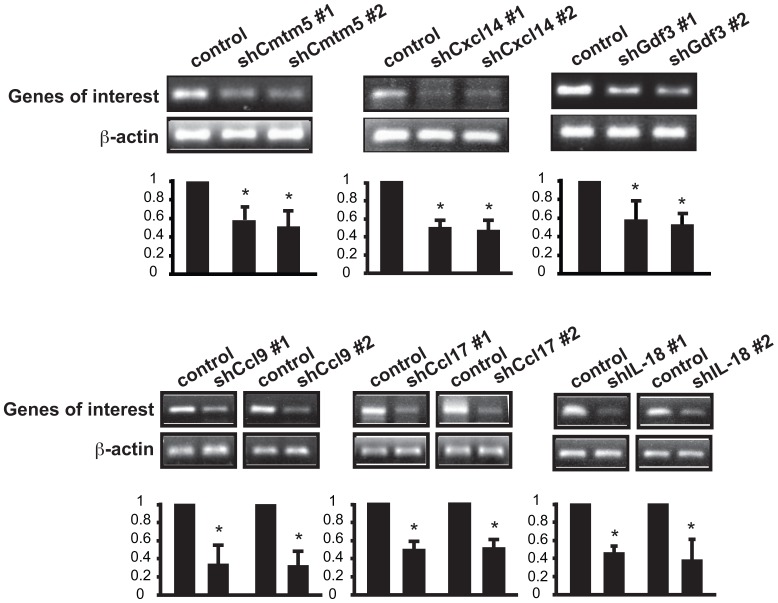
Validation of expression and knockdown of 6 additional candidate genes. C2C12 myoblasts were transduced overnight with lentiviruses expressing shRNAs as indicated. After 2-day puromycin selection, total RNA was extracted and subjected to RT-PCR. The results were quantified by densitometry and normalized to β-actin control. One sample t test was performed to compare each data point to control. Data shown are mean ± SD (n = 3). *P<0.05.

Six additional cytokines were confirmed for their RNAi knockdown efficiencies. They are Cmtm5, Cxcl14, and Gdf3 in class III, and Ccl9, Ccl17, and IL-18 in class IV (Fig. 7). Together, these cytokines represent a group of novel inhibitors of myoblast differentiation that may control the homeostasis of muscle formation.

### Conclusions

Our RNAi screen has revealed a diverse group of cytokines as potential regulators of myogenic differentiation. It is important to point out that this functional screen specifically uncovers myoblast/muscle-secreted cytokines that regulate myogenesis in a *cell-autonomous* fashion. While our study was in progress, several other cytokines were reported to modulate myogenic differentiation. Cardiotrophin-1 (CT-1) and Oncostatin M (OSM), both IL-6 family members, have been shown to suppress differentiation and muscle regeneration [41], [42]. On the other hand, granulocyte colony stimulating factor (G-CSF) stimulates myoblast proliferation and supports muscle regeneration [43]. It is not clear from the reports, however, whether any of those cytokines function cell-autonomously in skeletal muscle. All three genes were included in our RNAi screen; they did not make it to the positive hit list, but each had one shRNA eliciting a phenotype consistent with its reported myogenic function (data not shown). Deeper interrogation of the genes in the initial list (Table S1) with better shRNA coverage may reveal additional candidates for myogenic factors.

Together with the recent realization that numerous cytokines and chemokines are expressed in muscle cells [20], [21], [22], our findings suggest widespread involvement of these immunoregulatory molecules in myogenesis that is independent of their immunological functions. Further investigations will be necessary to uncover the cellular and molecular pathways by which these cytokines function in myogenesis, and may reveal novel therapeutic targets for improving muscle regeneration.

## Supporting Information

Table S1 A list of cytokine genes and their shRNAs covered in RNAi screen.
(DOCX)

Table S2 Gene-specific primers for RT-PCR.
(DOCX)

Table S3 Quantification of myotube formation for 29 candidate genes.
(DOCX)
