# Supplementary material for: RNAi Screen Reveals Potentially Novel Roles of Cytokines in Myoblast Differentiation
Source: PLoS One. 2013 Jul 2;8(7):e68068. doi: 10.1371/journal.pone.0068068 (PMC3699544; doi:10.1371/journal.pone.0068068)
Supplement: Table S1 — A list of cytokine genes and their shRNAs covered in RNAi screen. (DOCX) [file pone.0068068.s001.docx]

**Table S1. List of cytokine genes and their shRNAs covered in RNAi screen.**

| **Symbol** | **Gene Description** | **Clone ID** | **Clone Name** | **hairpin Sequence** |
| --- | --- | --- | --- | --- |
| A2m | alpha-2-macroglobulin | TRCN0000080503 | NM_175628.2-1511s1c1 | CCGGCCAGGCTCATTATATTCTAAACTCGAGTTTAGAATATAATGAGCCTGGTTTTTG |
| A2m | alpha-2-macroglobulin | TRCN0000080504 | NM_175628.2-3091s1c1 | CCGGCGGGTTACCAAAGACAATTAACTCGAGTTAATTGTCTTTGGTAACCCGTTTTTG |
| A2m | alpha-2-macroglobulin | TRCN0000080505 | NM_175628.2-230s1c1 | CCGGCCTCCTGTTCAACCACCTAAACTCGAGTTTAGGTGGTTGAACAGGAGGTTTTTG |
| A2m | alpha-2-macroglobulin | TRCN0000080506 | NM_175628.2-4031s1c1 | CCGGCCTCCAGACATCCTTGAAATACTCGAGTATTTCAAGGATGTCTGGAGGTTTTTG |
| A2m | alpha-2-macroglobulin | TRCN0000080507 | NM_175628.2-2093s1c1 | CCGGCCTAAAGGATATGGGCTTAAACTCGAGTTTAAGCCCATATCCTTTAGGTTTTTG |
| Areg | amphiregulin | TRCN0000089050 | NM_009704.2-649s1c1 | CCGGCGAATGCAGATACATCGAGAACTCGAGTTCTCGATGTATCTGCATTCGTTTTTG |
| Areg | amphiregulin | TRCN0000089051 | NM_009704.2-449s1c1 | CCGGCCACAAATATCCGGCTATATTCTCGAGAATATAGCCGGATATTTGTGGTTTTTG |
| Ccl1 | chemokine (C-C motif) ligand 1 | TRCN0000077013 | NM_011329.1-206s1c1 | CCGGGTTTATCCAGTGTTACAGAAACTCGAGTTTCTGTAACACTGGATAAACTTTTTG |
| Ccl1 | chemokine (C-C motif) ligand 1 | TRCN0000077016 | NM_011329.1-114s1c1 | CCGGGCCGTGTGGATACAGGATGTTCTCGAGAACATCCTGTATCCACACGGCTTTTTG |
| Ccl1 | chemokine (C-C motif) ligand 1 | TRCN0000077017 | NM_011329.1-167s1c1 | CCGGCTGCTGCTTGAACACCTTGAACTCGAGTTCAAGGTGTTCAAGCAGCAGTTTTTG |
| Ccl11 | chemokine (C-C motif) ligand 11, Eotaxin | TRCN0000067458 | NM_011330.1-206s1c1 | CCGGCAACAACAGATGCACCCTGAACTCGAGTTCAGGGTGCATCTGTTGTTGTTTTTG |
| Ccl11 | chemokine (C-C motif) ligand 11, Eotaxin | TRCN0000067460 | NM_011330.1-176s1c1 | CCGGCACACTACTGAAGAGCTACAACTCGAGTTGTAGCTCTTCAGTAGTGTGTTTTTG |
| Ccl11 | chemokine (C-C motif) ligand 11, Eotaxin | TRCN0000067461 | NM_011330.1-297s1c1 | CCGGGCCACAAAGCACCTGGACCAACTCGAGTTGGTCCAGGTGCTTTGTGGCTTTTTG |
| Ccl11 | chemokine (C-C motif) ligand 11, Eotaxin | TRCN0000067459 | NM_011330.1-142s1c1 | CCGGGCTGCTTTATCATGACCAGTACTCGAGTACTGGTCATGATAAAGCAGCTTTTTG |
| Ccl11 | chemokine (C-C motif) ligand 11, Eotaxin | TRCN0000067462 | NM_011330.1-218s1c1 | CCGGCACCCTGAAAGCCATAGTCTTCTCGAGAAGACTATGGCTTTCAGGGTGTTTTTG |
| Ccl12 | chemokine (C-C motif) ligand 12 | TRCN0000067653 | NM_011331.1-206s1c1 | CCGGGCTGTGATCTTCAGGACCATACTCGAGTATGGTCCTGAAGATCACAGCTTTTTG |
| Ccl12 | chemokine (C-C motif) ligand 12 | TRCN0000067654 | NM_011331.1-117s1c1 | CCGGCGTGCTGTTATAATGTTGTTACTCGAGTAACAACATTATAACAGCACGTTTTTG |
| Ccl12 | chemokine (C-C motif) ligand 12 | TRCN0000067655 | NM_011331.1-63s1c1 | CCGGCCACCATCAGTCCTCAGGTATCTCGAGATACCTGAGGACTGATGGTGGTTTTTG |
| Ccl17 | chemokine (C-C motif) ligand 17 | TRCN0000067558 | NM_011332.2-166s1c1 | CCGGAGGAAGTTGGTGAGCTGGTATCTCGAGATACCAGCTCACCAACTTCCTTTTTTG |
| Ccl17 | chemokine (C-C motif) ligand 17 | TRCN0000067559 | NM_011332.2-279s1c1 | CCGGGAAGGCCATCAGATTGGTGAACTCGAGTTCACCAATCTGATGGCCTTCTTTTTG |
| Ccl17 | chemokine (C-C motif) ligand 17 | TRCN0000067560 | NM_011332.2-123s1c1 | CCGGCCGAGAGTGCTGCCTGGATTACTCGAGTAATCCAGGCAGCACTCTCGGTTTTTG |
| Ccl19 | chemokine (C-C motif) ligand 19 | TRCN0000067608 | NM_011888.2-376s1c1 | CCGGCTGTTGTGTTCACCACACTAACTCGAGTTAGTGTGGTGAACACAACAGTTTTTG |
| Ccl19 | chemokine (C-C motif) ligand 19 | TRCN0000067609 | NM_011888.2-461s1c1 | CCGGGAAGTCTTCTGCCAAGAACAACTCGAGTTGTTCTTGGCAGAAGACTTCTTTTTG |
| Ccl19 | chemokine (C-C motif) ligand 19 | TRCN0000067610 | NM_011888.2-339s1c1 | CCGGCGCTACCTTCTTAATGAAGATCTCGAGATCTTCATTAAGAAGGTAGCGTTTTTG |
| Ccl2 | chemokine (C-C motif) ligand 2, MCP1 | TRCN0000034471 | NM_011333.1-433s1c1 | CCGGGAATGTGAAGTTGACCCGTAACTCGAGTTACGGGTCAACTTCACATTCTTTTTG |
| Ccl2 | chemokine (C-C motif) ligand 2, MCP1 | TRCN0000034472 | NM_011333.1-190s1c1 | CCGGCTGCTACTCATTCACCAGCAACTCGAGTTGCTGGTGAATGAGTAGCAGTTTTTG |
| Ccl2 | chemokine (C-C motif) ligand 2, MCP1 | TRCN0000034473 | NM_011333.1-329s1c1 | CCGGGAATGGGTCCAGACATACATTCTCGAGAATGTATGTCTGGACCCATTCTTTTTG |
| Ccl2 | chemokine (C-C motif) ligand 2, MCP1 | TRCN0000034470 | NM_011333.1-207s1c1 | CCGGGCAAGATGATCCCAATGAGTACTCGAGTACTCATTGGGATCATCTTGCTTTTTG |
| Ccl2 | chemokine (C-C motif) ligand 2, MCP1 | TRCN0000034469 | NM_011333.1-362s1c1 | CCGGCGGAACCAAATGAGATCAGAACTCGAGTTCTGATCTCATTTGGTTCCGTTTTTG |
| Ccl20 | chemokine (C-C motif) ligand 20 | TRCN0000067704 | NM_016960.1-315s1c1 | CCGGCCTAAGAGTCAAGAAGATGTACTCGAGTACATCTTCTTGACTCTTAGGTTTTTG |
| Ccl20 | chemokine (C-C motif) ligand 20 | TRCN0000067706 | NM_016960.1-274s1c1 | CCGGCCAAAGCAGAACTGGGTGAAACTCGAGTTTCACCCAGTTCTGCTTTGGTTTTTG |
| Ccl21a | chemokine (C-C motif) ligand 21a | TRCN0000067788 | NM_011124.3-401s1c1 | CCGGCTAAGTCTGGAAAGAAAGGAACTCGAGTTCCTTTCTTTCCAGACTTAGTTTTTG |
| Ccl21a | chemokine (C-C motif) ligand 21a | TRCN0000067789 | NM_011124.3-217s1c1 | CCGGAGGAAGCAAGAACCAAGTTTACTCGAGTAAACTTGGTTCTTGCTTCCTTTTTTG |
| Ccl21a | chemokine (C-C motif) ligand 21a | TRCN0000067790 | NM_011124.3-195s1c1 | CCGGCTACAGTATTGTCCGAGGCTACTCGAGTAGCCTCGGACAATACTGTAGTTTTTG |
| Ccl21b | chemokine (C-C motif) ligand 21b | TRCN0000089509 | NM_011335.1-419s1c1 | CCGGCTGAACAGACACAGCCCTCAACTCGAGTTGAGGGCTGTGTCTGTTCAGTTTTTG |
| Ccl21b | chemokine (C-C motif) ligand 21b | TRCN0000089511 | NM_011335.1-380s1c1 | CCGGCTGGAAAGAAAGGAAAGGGCTCTCGAGAGCCCTTTCCTTTCTTTCCAGTTTTTG |
| Ccl21b | chemokine (C-C motif) ligand 21b | TRCN0000089512 | NM_011335.1-129s1c1 | CCGGACAGGACTGCTGCCTTAAGTACTCGAGTACTTAAGGCAGCAGTCCTGTTTTTTG |
| Ccl21b | chemokine (C-C motif) ligand 21b | TRCN0000089508 | NM_011335.1-594s1c1 | CCGGGCCCTGCTTCAACCATTACATCTCGAGATGTAATGGTTGAAGCAGGGCTTTTTG |
| Ccl21b | chemokine (C-C motif) ligand 21b | TRCN0000089510 | NM_011335.1-254s1c1 | CCGGCTAAGCCTGAGCTATGTGCAACTCGAGTTGCACATAGCTCAGGCTTAGTTTTTG |
| Ccl21c | chemokine (C-C motif) ligand 21c (leucine) | TRCN0000067748 | NM_023052.1-172s1c1 | CCGGCGAGGCTATAGGAAGCAAGAACTCGAGTTCTTGCTTCCTATAGCCTCGTTTTTG |
| Ccl21c | chemokine (C-C motif) ligand 21c (leucine) | TRCN0000067749 | NM_023052.1-132s1c1 | CCGGCCTTAAGTACAGCCAGAAGAACTCGAGTTCTTCTGGCTGTACTTAAGGTTTTTG |
| Ccl21c | chemokine (C-C motif) ligand 21c (leucine) | TRCN0000067750 | NM_023052.1-256s1c1 | CCGGCTATGTGCAAACCCTGAGGAACTCGAGTTCCTCAGGGTTTGCACATAGTTTTTG |
| Ccl22 | chemokine (C-C motif) ligand 22 | TRCN0000067833 | NM_009137.1-1041s1c1 | CCGGGCTCAGAATCAGATTTCTTAACTCGAGTTAAGAAATCTGATTCTGAGCTTTTTG |
| Ccl22 | chemokine (C-C motif) ligand 22 | TRCN0000067834 | NM_009137.1-380s1c1 | CCGGGCTACTCCATAAACTGTCCTACTCGAGTAGGACAGTTTATGGAGTAGCTTTTTG |
| Ccl22 | chemokine (C-C motif) ligand 22 | TRCN0000067835 | NM_009137.1-257s1c1 | CCGGGCCATCACGTTTAGTGAAGGACTCGAGTCCTTCACTAAACGTGATGGCTTTTTG |
| Ccl24 | chemokine (C-C motif) ligand 24 | TRCN0000067878 | NM_019577.2-248s1c1 | CCGGCATCACCAAGAAGGGCCATAACTCGAGTTATGGCCCTTCTTGGTGATGTTTTTG |
| Ccl24 | chemokine (C-C motif) ligand 24 | TRCN0000067879 | NM_019577.2-156s1c1 | CCGGACGTCCTTTATTTCCAAGAAACTCGAGTTTCTTGGAAATAAAGGACGTTTTTTG |
| Ccl24 | chemokine (C-C motif) ligand 24 | TRCN0000067880 | NM_019577.2-390s1c1 | CCGGCGTGGCAATAGCACCGAGGTTCTCGAGAACCTCGGTGCTATTGCCACGTTTTTG |
| Ccl25 | chemokine (C-C motif) ligand 25 | TRCN0000067915 | NM_009138.1-295s1c1 | CCGGCCAGAAAGTAGTGTGTGGGAACTCGAGTTCCCACACACTACTTTCTGGTTTTTG |
| Ccl25 | chemokine (C-C motif) ligand 25 | TRCN0000067917 | NM_009138.1-281s1c1 | CCGGAGATTCTACTTCCGCCAGAAACTCGAGTTTCTGGCGGAAGTAGAATCTTTTTTG |
| Ccl28 | chemokine (C-C motif) ligand 28 | TRCN0000065743 | NM_020279.2-241s1c1 | CCGGGCTGTCATCCTTCATGTTAAACTCGAGTTTAACATGAAGGATGACAGCTTTTTG |
| Ccl28 | chemokine (C-C motif) ligand 28 | TRCN0000065744 | NM_020279.2-282s1c1 | CCGGCCCGCACAATCGTACTTTGAACTCGAGTTCAAAGTACGATTGTGCGGGTTTTTG |
| Ccl28 | chemokine (C-C motif) ligand 28 | TRCN0000065745 | NM_020279.2-152s1c1 | CCGGCTGAGGTGTCTCATCATGTTTCTCGAGAAACATGATGAGACACCTCAGTTTTTG |
| Ccl3 | chemokine (C-C motif) ligand 3 | TRCN0000068003 | NM_011337.1-210s1c1 | CCGGCGCCAATTCATCGTTGACTATCTCGAGATAGTCAACGATGAATTGGCGTTTTTG |
| Ccl3 | chemokine (C-C motif) ligand 3 | TRCN0000068004 | NM_011337.1-196s1c1 | CCGGGCCGGAAGATTCCACGCCAATCTCGAGATTGGCGTGGAATCTTCCGGCTTTTTG |
| Ccl3 | chemokine (C-C motif) ligand 3 | TRCN0000068005 | NM_011337.1-275s1c1 | CCGGGACTAAGAGAAACCGGCAGATCTCGAGATCTGCCGGTTTCTCTTAGTCTTTTTG |
| Ccl4 | chemokine (C-C motif) ligand 4 | TRCN0000068048 | NM_013652.1-408s1c1 | CCGGCCTGATGCTTCTCACTGAGAACTCGAGTTCTCAGTGAGAAGCATCAGGTTTTTG |
| Ccl4 | chemokine (C-C motif) ligand 4 | TRCN0000068049 | NM_013652.1-261s1c1 | CCGGGCTGTGGTATTCCTGACCAAACTCGAGTTTGGTCAGGAATACCACAGCTTTTTG |
| Ccl4 | chemokine (C-C motif) ligand 4 | TRCN0000068050 | NM_013652.1-203s1c1 | CCGGGCTTCACAGAAGCTTTGTGATCTCGAGATCACAAAGCTTCTGTGAAGCTTTTTG |
| Ccl5 | chemokine (C-C motif) ligand 5 | TRCN0000068098 | NM_013653.1-327s1c1 | CCGGTCTTGATTCTGACCCTGTATACTCGAGTATACAGGGTCAGAATCAAGATTTTTG |
| Ccl5 | chemokine (C-C motif) ligand 5 | TRCN0000068099 | NM_013653.1-265s1c1 | CCGGCCAGAGAAGAAGTGGGTTCAACTCGAGTTGAACCCACTTCTTCTCTGGTTTTTG |
| Ccl5 | chemokine (C-C motif) ligand 5 | TRCN0000068100 | NM_013653.1-225s1c1 | CCGGCGTGTTTGTCACTCGAAGGAACTCGAGTTCCTTCGAGTGACAAACACGTTTTTG |
| Ccl6 | chemokine (C-C motif) ligand 6 | TRCN0000089328 | NM_009139.1-701s1c1 | CCGGCGGGTAATATCTAGCTGAGATCTCGAGATCTCAGCTAGATATTACCCGTTTTTG |
| Ccl6 | chemokine (C-C motif) ligand 6 | TRCN0000089329 | NM_009139.1-143s1c1 | CCGGCGTCGCTATAACCCTCCAATACTCGAGTATTGGAGGGTTATAGCGACGTTTTTG |
| Ccl6 | chemokine (C-C motif) ligand 6 | TRCN0000089330 | NM_009139.1-114s1c1 | CCGGGCCTCATACAAGAAATGGAAACTCGAGTTTCCATTTCTTGTATGAGGCTTTTTG |
| Ccl7 | chemokine (C-C motif) ligand 7 | TRCN0000068135 | NM_013654.2-313s1c1 | CCGGCGAGGAGGCTATAGCATACTTCTCGAGAAGTATGCTATAGCCTCCTCGTTTTTG |
| Ccl7 | chemokine (C-C motif) ligand 7 | TRCN0000068136 | NM_013654.2-110s1c1 | CCGGGCCGCTGCTTTCAGCATCCAACTCGAGTTGGATGCTGAAAGCAGCGGCTTTTTG |
| Ccl8 | chemokine (C-C motif) ligand 8 | TRCN0000068173 | NM_021443.1-322s1c1 | CCGGCTTGACCAGAAGTCTCAAATTCTCGAGAATTTGAGACTTCTGGTCAAGTTTTTG |
| Ccl8 | chemokine (C-C motif) ligand 8 | TRCN0000068176 | NM_021443.1-207s1c1 | CCGGCGAGAGAATCAACAATATCCACTCGAGTGGATATTGTTGATTCTCTCGTTTTTG |
| Ccl8 | chemokine (C-C motif) ligand 8 | TRCN0000068174 | NM_021443.1-162s1c1 | CCGGTCATGTACTAAAGCTGAAGATCTCGAGATCTTCAGCTTTAGTACATGATTTTTG |
| Ccl8 | chemokine (C-C motif) ligand 8 | TRCN0000068177 | NM_021443.1-312s1c1 | CCGGCATGGAGATCCTTGACCAGAACTCGAGTTCTGGTCAAGGATCTCCATGTTTTTG |
| Ccl9 | chemokine (C-C motif) ligand 9 | TRCN0000077008 | NM_011338.1-915s1c1 | CCGGGCCCTTTAGTTAGTAGTATTTCTCGAGAAATACTACTAACTAAAGGGCTTTTTG |
| Ccl9 | chemokine (C-C motif) ligand 9 | TRCN0000077009 | NM_011338.1-327s1c1 | CCGGCCTGTCCTATAACTCACGGATCTCGAGATCCGTGAGTTATAGGACAGGTTTTTG |
| Ccl9 | chemokine (C-C motif) ligand 9 | TRCN0000077010 | NM_011338.1-457s1c1 | CCGGCGGAGAGTTCAGAGATGCATTCTCGAGAATGCATCTCTGAACTCTCCGTTTTTG |
| Cd40lg | CD40 ligand | TRCN0000066583 | NM_011616.2-871s1c1 | CCGGCCTGTGTTGAACTGCCTATTTCTCGAGAAATAGGCAGTTCAACACAGGTTTTTG |
| Cd40lg | CD40 ligand | TRCN0000066584 | NM_011616.2-286s1c1 | CCGGGAAGACCTTGTCAAGGATATACTCGAGTATATCCTTGACAAGGTCTTCTTTTTG |
| Cd40lg | CD40 ligand | TRCN0000066585 | NM_011616.2-426s1c1 | CCGGGTGGGCCAAGAAAGGATATTACTCGAGTAATATCCTTTCTTGGCCCACTTTTTG |
| Cer1 | cerberus 1 homolog (Xenopus laevis) | TRCN0000098175 | NM_009887.1-1017s1c1 | CCGGCCCTCTCTCTTCTCTCTATTTCTCGAGAAATAGAGAGAAGAGAGAGGGTTTTTG |
| Cer1 | cerberus 1 homolog (Xenopus laevis) | TRCN0000098176 | NM_009887.1-484s1c1 | CCGGGCATCGGTTCATGTTCAGAAACTCGAGTTTCTGAACATGAACCGATGCTTTTTG |
| Cer1 | cerberus 1 homolog (Xenopus laevis) | TRCN0000098177 | NM_009887.1-640s1c1 | CCGGCTTTGGCAAATGCAGTTCCATCTCGAGATGGAACTGCATTTGCCAAAGTTTTTG |
| Cklf | chemokine-like factor, CKLF | TRCN0000089298 | NM_029295.1-308s1c1 | CCGGCGTGTGGACTTGACAAGATAACTCGAGTTATCTTGTCAAGTCCACACGTTTTTG |
| Cklf | chemokine-like factor, CKLF | TRCN0000089299 | NM_029295.1-462s1c1 | CCGGCCTGACAGTAACATGTACTATCTCGAGATAGTACATGTTACTGTCAGGTTTTTG |
| Cklf | chemokine-like factor, CKLF | TRCN0000089300 | NM_029295.1-152s1c1 | CCGGCCTTCTGCTGTACTCTGAAATCTCGAGATTTCAGAGTACAGCAGAAGGTTTTTG |
| Cklf | chemokine-like factor, CKLF | TRCN0000089301 | NM_029295.1-307s1c1 | CCGGACGTGTGGACTTGACAAGATACTCGAGTATCTTGTCAAGTCCACACGTTTTTTG |
| Cklf | chemokine-like factor, CKLF | TRCN0000089302 | NM_029295.1-397s1c1 | CCGGGTGTCTGTGTTGGCTCTAATACTCGAGTATTAGAGCCAACACAGACACTTTTTG |
| Clcf1 | cardiotrophin-like cytokine factor 1, CLCF1 | TRCN0000088928 | NM_019952.1-173s1c1 | CCGGGCTCTTAATCGCACAGGAGATCTCGAGATCTCCTGTGCGATTAAGAGCTTTTTG |
| Clcf1 | cardiotrophin-like cytokine factor 1, CLCF1 | TRCN0000088930 | NM_019952.1-628s1c1 | CCGGCCTCCAGAAGATGGATGACTTCTCGAGAAGTCATCCATCTTCTGGAGGTTTTTG |
| Clcf1 | cardiotrophin-like cytokine factor 1, CLCF1 | TRCN0000088931 | NM_019952.1-468s1c1 | CCGGCCACAGCTGAACTCCGACGTACTCGAGTACGTCGGAGTTCAGCTGTGGTTTTTG |
| Clcf1 | cardiotrophin-like cytokine factor 1, CLCF1 | TRCN0000088929 | NM_019952.1-360s1c1 | CCGGTCAACTTGGAAGTGTGGCGAACTCGAGTTCGCCACACTTCCAAGTTGATTTTTG |
| Clcf1 | cardiotrophin-like cytokine factor 1, CLCF1 | TRCN0000088932 | NM_019952.1-295s1c1 | CCGGCCCTTTCAACGAGCCTGACTTCTCGAGAAGTCAGGCTCGTTGAAAGGGTTTTTG |
| Cmtm2a | CKLF-like MARVEL transmembrane domain containing 2A | TRCN0000241352 | NM_027022.4-474s21c1 | CCGGTTCCTTGGAGGTGGCATATACCTCGAGGTATATGCCACCTCCAAGGAATTTTTG |
| Cmtm2a | CKLF-like MARVEL transmembrane domain containing 2A | TRCN0000241353 | NM_027022.4-803s21c1 | CCGGCAACCTTAAACCTTAACTAACCTCGAGGTTAGTTAAGGTTTAAGGTTGTTTTTG |
| Cmtm2a | CKLF-like MARVEL transmembrane domain containing 2A | TRCN0000241354 | NM_027022.4-578s21c1 | CCGGCATCGACATGTTACTTCAATTCTCGAGAATTGAAGTAACATGTCGATGTTTTTG |
| Cmtm2b | CKLF-like MARVEL transmembrane domain containing 2B | TRCN0000246548 | NM_028524.2-317s21c1 | CCGGGTAATCCTGCTGCTTACTATGCTCGAGCATAGTAAGCAGCAGGATTACTTTTTG |
| Cmtm2b | CKLF-like MARVEL transmembrane domain containing 2B | TRCN0000246549 | NM_028524.2-601s21c1 | CCGGTCGAAAGTTTATTCTACTTACCTCGAGGTAAGTAGAATAAACTTTCGATTTTTG |
| Cmtm2b | CKLF-like MARVEL transmembrane domain containing 2B | TRCN0000246550 | NM_028524.2-452s21c1 | CCGGTTCCTCATAGGTGGTATATTCCTCGAGGAATATACCACCTATGAGGAATTTTTG |
| Cmtm3 | CKLF-like MARVEL transmembrane domain containing 3 | TRCN0000090318 | NM_024217.2-1402s1c1 | CCGGGCGGTTACTGTGTGTGTCTTTCTCGAGAAAGACACACACAGTAACCGCTTTTTG |
| Cmtm3 | CKLF-like MARVEL transmembrane domain containing 3 | TRCN0000090319 | NM_024217.2-564s1c1 | CCGGGCCACAATTGTGTTCGCAATTCTCGAGAATTGCGAACACAATTGTGGCTTTTTG |
| Cmtm3 | CKLF-like MARVEL transmembrane domain containing 3 | TRCN0000090320 | NM_024217.2-381s1c1 | CCGGGCCGTTTACTTCCTCTTTGCTCTCGAGAGCAAAGAGGAAGTAAACGGCTTTTTG |
| Cmtm3 | CKLF-like MARVEL transmembrane domain containing 3 | TRCN0000090322 | NM_024217.2-612s1c1 | CCGGGCCAAATTCCTCAAGCAAGGACTCGAGTCCTTGCTTGAGGAATTTGGCTTTTTG |
| Cmtm3 | CKLF-like MARVEL transmembrane domain containing 3 | TRCN0000090321 | NM_024217.2-295s1c1 | CCGGCGGGTCTTTCATTCATCACCTCTCGAGAGGTGATGAATGAAAGACCCGTTTTTG |
| Cmtm4 | CKLF-like MARVEL transmembrane domain containing 4 | TRCN0000126359 | NM_153582.3-908s1c1 | CCGGCCGACTTTAATGACTGCTCTACTCGAGTAGAGCAGTCATTAAAGTCGGTTTTTG |
| Cmtm4 | CKLF-like MARVEL transmembrane domain containing 4 | TRCN0000126360 | NM_153582.3-506s1c1 | CCGGCGGAAATTGCTGCCGTGATATCTCGAGATATCACGGCAGCAATTTCCGTTTTTG |
| Cmtm4 | CKLF-like MARVEL transmembrane domain containing 4 | TRCN0000126361 | NM_153582.3-401s1c1 | CCGGCCCAGATCAACTGGAATCTAACTCGAGTTAGATTCCAGTTGATCTGGGTTTTTG |
| Cmtm5 | CKLF-like MARVEL transmembrane domain containing 5 | TRCN0000090798 | NM_026066.1-707s1c1 | CCGGCCCATCTTTGTCATCTTTGAACTCGAGTTCAAAGATGACAAAGATGGGTTTTTG |
| Cmtm5 | CKLF-like MARVEL transmembrane domain containing 5 | TRCN0000090799 | NM_026066.1-579s1c1 | CCGGGTCTCCGTCTTTGCCTATGATCTCGAGATCATAGGCAAAGACGGAGACTTTTTG |
| Cmtm5 | CKLF-like MARVEL transmembrane domain containing 5 | TRCN0000090800 | NM_026066.1-608s1c1 | CCGGGATCTACCGAACTGAGCTGATCTCGAGATCAGCTCAGTTCGGTAGATCTTTTTG |
| Cmtm6 | CKLF-like MARVEL transmembrane domain containing 6 | TRCN0000121352 | NM_026036.1-963s1c1 | CCGGCCGGAGATTTAATGAGTGTTTCTCGAGAAACACTCATTAAATCTCCGGTTTTTG |
| Cmtm6 | CKLF-like MARVEL transmembrane domain containing 6 | TRCN0000121354 | NM_026036.1-426s1c1 | CCGGCCTCAGCTGAAATTGCTGCAACTCGAGTTGCAGCAATTTCAGCTGAGGTTTTTG |
| Cmtm6 | CKLF-like MARVEL transmembrane domain containing 6 | TRCN0000121355 | NM_026036.1-526s1c1 | CCGGGCTGAGAAAGCCCGAGAACAACTCGAGTTGTTCTCGGGCTTTCTCAGCTTTTTG |
| Cmtm7 | CKLF-like MARVEL transmembrane domain containing 7 | TRCN0000127226 | NM_133978.1-345s1c1 | CCGGGCACTATTTAATCGGCACGCTCTCGAGAGCGTGCCGATTAAATAGTGCTTTTTG |
| Cmtm7 | CKLF-like MARVEL transmembrane domain containing 7 | TRCN0000127227 | NM_133978.1-383s1c1 | CCGGCCATAGTGATAGCCTCCAAGACTCGAGTCTTGGAGGCTATCACTATGGTTTTTG |
| Cmtm7 | CKLF-like MARVEL transmembrane domain containing 7 | TRCN0000127228 | NM_133978.1-494s1c1 | CCGGTCACCTGTATAACCCAGTCTTCTCGAGAAGACTGGGTTATACAGGTGATTTTTG |
| Cmtm8 | CKLF-like MARVEL transmembrane domain containing 8 | TRCN0000104920 | NM_027294.1-783s1c1 | CCGGCCAGAACGAATCCCACTGTTACTCGAGTAACAGTGGGATTCGTTCTGGTTTTTG |
| Cmtm8 | CKLF-like MARVEL transmembrane domain containing 8 | TRCN0000104921 | NM_027294.1-503s1c1 | CCGGACCCTGACTTACACCAGGATTCTCGAGAATCCTGGTGTAAGTCAGGGTTTTTTG |
| Cmtm8 | CKLF-like MARVEL transmembrane domain containing 8 | TRCN0000104922 | NM_027294.1-691s1c1 | CCGGCTATGCTGGAAACACGTACTTCTCGAGAAGTACGTGTTTCCAGCATAGTTTTTG |
| Cntf | ciliary neurotrophic factor | TRCN0000065813 | NM_170786.1-597s1c1 | CCGGCCTACCAGCTAGAGGAGTTAACTCGAGTTAACTCCTCTAGCTGGTAGGTTTTTG |
| Cntf | ciliary neurotrophic factor | TRCN0000065814 | NM_170786.1-335s1c1 | CCGGGCTCTTATGGAATCTTATGTACTCGAGTACATAAGATTCCATAAGAGCTTTTTG |
| Csf1 | Macrophage colony-stimulating factor, M-CSF | TRCN0000065908 | NM_007778.1-1940s1c1 | CCGGGCCTACCAAGACTGGATGAAACTCGAGTTTCATCCAGTCTTGGTAGGCTTTTTG |
| Csf1 | Macrophage colony-stimulating factor, M-CSF | TRCN0000065911 | NM_007778.1-1692s1c1 | CCGGCCTCCTGTTCTACAAGTGGAACTCGAGTTCCACTTGTAGAACAGGAGGTTTTTG |
| Csf1 | Macrophage colony-stimulating factor, M-CSF | TRCN0000065912 | NM_007778.1-344s1c1 | CCGGCATGCCAGATTGCCTTTGAATCTCGAGATTCAAAGGCAATCTGGCATGTTTTTG |
| Csf1 | Macrophage colony-stimulating factor, M-CSF | TRCN0000065909 | NM_007778.1-1411s1c1 | CCGGGCTCAGTTACTGCTTCCCAAACTCGAGTTTGGGAAGCAGTAACTGAGCTTTTTG |
| Csf1 | Macrophage colony-stimulating factor, M-CSF | TRCN0000065910 | NM_007778.1-942s1c1 | CCGGCGAGTCAACAGAGCAACCAAACTCGAGTTTGGTTGCTCTGTTGACTCGTTTTTG |
| Csf2 | Granulocyte-macrophage colony-stimulating factor, GM-CSF | TRCN0000054618 | NM_009969.2-385s1c1 | CCGGCGGATTTCATAGACAGCCTTACTCGAGTAAGGCTGTCTATGAAATCCGTTTTTG |
| Csf2 | Granulocyte-macrophage colony-stimulating factor, GM-CSF | TRCN0000054619 | NM_009969.2-200s1c1 | CCGGCGTCTCTAACGAGTTCTCCTTCTCGAGAAGGAGAACTCGTTAGAGACGTTTTTG |
| Csf2 | Granulocyte-macrophage colony-stimulating factor, GM-CSF | TRCN0000054620 | NM_009969.2-314s1c1 | CCGGAGCCAGCTACTACCAGACATACTCGAGTATGTCTGGTAGTAGCTGGCTTTTTTG |
| Csf2 | Granulocyte-macrophage colony-stimulating factor, GM-CSF | TRCN0000054621 | NM_009969.2-170s1c1 | CCGGGCCTGTCACATTGAATGAAGACTCGAGTCTTCATTCAATGTGACAGGCTTTTTG |
| Csf2 | Granulocyte-macrophage colony-stimulating factor, GM-CSF | TRCN0000054622 | NM_009969.2-122s1c1 | CCGGGAAGCATGTAGAGGCCATCAACTCGAGTTGATGGCCTCTACATGCTTCTTTTTG |
| Csf3 | Granulocyte colony-stimulating factor, G-CSF | TRCN0000066008 | NM_009971.1-441s1c1 | CCGGTGCAGGCTCTATCGGGTATTTCTCGAGAAATACCCGATAGAGCCTGCATTTTTG |
| Csf3 | Granulocyte colony-stimulating factor, G-CSF | TRCN0000066009 | NM_009971.1-494s1c1 | CCGGCAGCTGGATGTTGCCAACTTTCTCGAGAAAGTTGGCAACATCCAGCTGTTTTTG |
| Csf3 | Granulocyte colony-stimulating factor, G-CSF | TRCN0000066010 | NM_009971.1-225s1c1 | CCGGCCCTGGAGCAAGTGAGGAAGACTCGAGTCTTCCTCACTTGCTCCAGGGTTTTTG |
| Csf3 | Granulocyte colony-stimulating factor, G-CSF | TRCN0000066011 | NM_009971.1-628s1c1 | CCGGCCTGGCCATTTCGTACCTGCACTCGAGTGCAGGTACGAAATGGCCAGGTTTTTG |
| Csf3 | Granulocyte colony-stimulating factor, G-CSF | TRCN0000066012 | NM_009971.1-274s1c1 | CCGGGCAGTTGTGTGCCACCTACAACTCGAGTTGTAGGTGGCACACAACTGCTTTTTG |
| Ctf1 | cardiotrophin 1, CT-1 | TRCN0000066059 | NM_007795.1-125s1c1 | CCGGCTCCTGACCAAATATGCAGAACTCGAGTTCTGCATATTTGGTCAGGAGTTTTTG |
| Ctf1 | cardiotrophin 1, CT-1 | TRCN0000066062 | NM_007795.1-490s1c1 | CCGGCGCCACCCTCTTCACGGCCAACTCGAGTTGGCCGTGAAGAGGGTGGCGTTTTTG |
| Ctf1 | cardiotrophin 1, CT-1 | TRCN0000066058 | NM_007795.1-140s1c1 | CCGGGCAGAACAACTTCTGGAGGAACTCGAGTTCCTCCAGAAGTTGTTCTGCTTTTTG |
| Ctf1 | cardiotrophin 1, CT-1 | TRCN0000066060 | NM_007795.1-49s1c1 | CCGGCCACCAGACTGACTCCTCAATCTCGAGATTGAGGAGTCAGTCTGGTGGTTTTTG |
| Ctf1 | cardiotrophin 1, CT-1 | TRCN0000066061 | NM_007795.1-559s1c1 | CCGGCGGCCTCTATGGCGAGTGGGTCTCGAGACCCACTCGCCATAGAGGCCGTTTTTG |
| Ctf2 | cardiotrophin 2 | TRCN0000190485 | NM_198858.1-371s1c1 | CCGGGACGACCAGAGTTATCTGAATCTCGAGATTCAGATAACTCTGGTCGTCTTTTTTG |
| Ctf2 | cardiotrophin 2 | TRCN0000190887 | NM_198858.1-628s1c1 | CCGGCAAGGCTAAGTACTCAGCATACTCGAGTATGCTGAGTACTTAGCCTTGTTTTTTG |
| Cx3cl1 | chemokine (C-X3-C motif) ligand 1 | TRCN0000065505 | NM_009142.2-120s1c1 | CCGGCGGCATGACGAAATGCGAAATCTCGAGATTTCGCATTTCGTCATGCCGTTTTTG |
| Cx3cl1 | chemokine (C-X3-C motif) ligand 1 | TRCN0000065506 | NM_009142.2-579s1c1 | CCGGGCCTCAGAGCATTGGAAGTTTCTCGAGAAACTTCCAATGCTCTGAGGCTTTTTG |
| Cx3cl1 | chemokine (C-X3-C motif) ligand 1 | TRCN0000065507 | NM_009142.2-827s1c1 | CCGGAGGAGATAAACCCAGTTCATACTCGAGTATGAACTGGGTTTATCTCCTTTTTTG |
| Cxcl1 | chemokine (C-X-C motif) ligand 1, KC | TRCN0000067209 | NM_008176.1-130s1c1 | CCGGCCTCAAGAACATCCAGAGCTTCTCGAGAAGCTCTGGATGTTCTTGAGGTTTTTG |
| Cxcl1 | chemokine (C-X-C motif) ligand 1, KC | TRCN0000067210 | NM_008176.1-107s1c1 | CCGGCTGCAGACCATGGCTGGGATTCTCGAGAATCCCAGCCATGGTCTGCAGTTTTTG |
| Cxcl1 | chemokine (C-X-C motif) ligand 1, KC | TRCN0000067212 | NM_008176.1-236s1c1 | CCGGGAAGCTCCCTTGGTTCAGAAACTCGAGTTTCTGAACCAAGGGAGCTTCTTTTTG |
| Cxcl1 | chemokine (C-X-C motif) ligand 1, KC | TRCN0000067208 | NM_008176.1-654s1c1 | CCGGGCTGTGTTTGTATGTCTTGAACTCGAGTTCAAGACATACAAACACAGCTTTTTG |
| Cxcl1 | chemokine (C-X-C motif) ligand 1, KC | TRCN0000067211 | NM_008176.1-124s1c1 | CCGGGATTCACCTCAAGAACATCCACTCGAGTGGATGTTCTTGAGGTGAATCTTTTTG |
| Cxcl10 | chemokine (C-X-C motif) ligand 10 | TRCN0000068208 | NM_021274.1-895s1c1 | CCGGGCATTGTATATGGAAGAACTTCTCGAGAAGTTCTTCCATATACAATGCTTTTTG |
| Cxcl10 | chemokine (C-X-C motif) ligand 10 | TRCN0000068209 | NM_021274.1-124s1c1 | CCGGCCGCTGCAACTGCATCCATATCTCGAGATATGGATGCAGTTGCAGCGGTTTTTG |
| Cxcl10 | chemokine (C-X-C motif) ligand 10 | TRCN0000068212 | NM_021274.1-268s1c1 | CCGGTCCGGAATCTAAGACCATCAACTCGAGTTGATGGTCTTAGATTCCGGATTTTTG |
| Cxcl11 | chemokine (C-X-C motif) ligand 11 | TRCN0000068243 | NM_019494.1-310s1c1 | CCGGCGCCTCATAATGCAGGCAATACTCGAGTATTGCCTGCATTATGAGGCGTTTTTG |
| Cxcl11 | chemokine (C-X-C motif) ligand 11 | TRCN0000068244 | NM_019494.1-206s1c1 | CCGGCTTCTGTAATTTACCCGAGTACTCGAGTACTCGGGTAAATTACAGAAGTTTTTG |
| Cxcl11 | chemokine (C-X-C motif) ligand 11 | TRCN0000068245 | NM_019494.1-253s1c1 | CCGGGTTACTATGAAGGCTCATAAACTCGAGTTTATGAGCCTTCATAGTAACTTTTTG |
| Cxcl12 | chemokine (C-X-C motif) ligand 12 | TRCN0000184347 | NM_013655.2-315s1c1 | CCGGGATCCAAGAGTACCTGGAGAACTCGAGTTCTCCAGGTACTCTTGGATCTTTTTTG |
| Cxcl12 | chemokine (C-X-C motif) ligand 12 | TRCN0000196073 | NM_013655.2-205s1c1 | CCGGGCCAACGTCAAGCATCTGAAACTCGAGTTTCAGATGCTTGACGTTGGCTTTTTTG |
| Cxcl12 | chemokine (C-X-C motif) ligand 12 | TRCN0000195944 | NM_013655.2-132s1c1 | CCGGCATCAGTGACGGTAAACCAGTCTCGAGACTGGTTTACCGTCACTGATGTTTTTTG |
| Cxcl12 | chemokine (C-X-C motif) ligand 12 | TRCN0000178772 | NM_013655.2-268s1c1 | CCGGCTGAAGAACAACAACAGACAACTCGAGTTGTCTGTTGTTGTTCTTCAGTTTTTTG |
| Cxcl12 | chemokine (C-X-C motif) ligand 12 | TRCN0000183874 | NM_013655.2-319s1c1 | CCGGCAAGAGTACCTGGAGAAAGCTCTCGAGAGCTTTCTCCAGGTACTCTTGTTTTTTG |
| Cxcl13 | chemokine (C-X-C motif) ligand 13 | TRCN0000068278 | NM_018866.1-270s1c1 | CCGGGCCAAATGGTTACAAAGATTACTCGAGTAATCTTTGTAACCATTTGGCTTTTTG |
| Cxcl13 | chemokine (C-X-C motif) ligand 13 | TRCN0000068279 | NM_018866.1-162s1c1 | CCGGCTAAACATCATAGATCGGATTCTCGAGAATCCGATCTATGATGTTTAGTTTTTG |
| Cxcl13 | chemokine (C-X-C motif) ligand 13 | TRCN0000068280 | NM_018866.1-102s1c1 | CCGGGAAGCCCATTACACAAACTTACTCGAGTAAGTTTGTGTAATGGGCTTCTTTTTG |
| Cxcl14 | chemokine (C-X-C motif) ligand 14 | TRCN0000065368 | NM_019568.1-628s1c1 | CCGGCAAGTGGTACAATGCCTGGAACTCGAGTTCCAGGCATTGTACCACTTGTTTTTG |
| Cxcl14 | chemokine (C-X-C motif) ligand 14 | TRCN0000065369 | NM_019568.1-526s1c1 | CCGGCTGCGAGGAGAAGATGGTTATCTCGAGATAACCATCTTCTCCTCGCAGTTTTTG |
| Cxcl14 | chemokine (C-X-C motif) ligand 14 | TRCN0000065370 | NM_019568.1-610s1c1 | CCGGGAGCACCAAACGCTTCATCAACTCGAGTTGATGAAGCGTTTGGTGCTCTTTTTG |
| Cxcl15 | chemokine (C-X-C motif) ligand 15 | TRCN0000112211 | NM_011339.1-259s1c1 | CCGGCCAATTACTAACAGGTTCCTACTCGAGTAGGAACCTGTTAGTAATTGGTTTTTG |
| Cxcl15 | chemokine (C-X-C motif) ligand 15 | TRCN0000112212 | NM_011339.1-258s1c1 | CCGGCCCAATTACTAACAGGTTCCTCTCGAGAGGAACCTGTTAGTAATTGGGTTTTTG |
| Cxcl15 | chemokine (C-X-C motif) ligand 15 | TRCN0000112213 | NM_011339.1-159s1c1 | CCGGGACCATTTACTGCAACAGAAACTCGAGTTTCTGTTGCAGTAAATGGTCTTTTTG |
| Cxcl16 | chemokine (C-X-C motif) ligand 16 | TRCN0000065695 | NM_023158.3-180s1c1 | CCGGGCTGGAAGTTGTTCTTGTGATCTCGAGATCACAAGAACAACTTCCAGCTTTTTG |
| Cxcl16 | chemokine (C-X-C motif) ligand 16 | TRCN0000065696 | NM_023158.3-618s1c1 | CCGGGAGGCAAATGAGAAACAGCAACTCGAGTTGCTGTTTCTCATTTGCCTCTTTTTG |
| Cxcl2 | chemokine (C-X-C motif) ligand 2 | TRCN0000067258 | NM_009140.1-172s1c1 | CCGGGCCAAGGGTTGACTTCAAGAACTCGAGTTCTTGAAGTCAACCCTTGGCTTTTTG |
| Cxcl2 | chemokine (C-X-C motif) ligand 2 | TRCN0000067259 | NM_009140.1-252s1c1 | CCGGCCACTCTCAAGGGCGGTCAAACTCGAGTTTGACCGCCCTTGAGAGTGGTTTTTG |
| Cxcl2 | chemokine (C-X-C motif) ligand 2 | TRCN0000067260 | NM_009140.1-325s1c1 | CCGGACTGAACAAAGGCAAGGCTAACTCGAGTTAGCCTTGCCTTTGTTCAGTTTTTTG |
| Cxcl5 | chemokine (C-X-C motif) ligand 5 | TRCN0000054598 | NM_009141.1-254s1c1 | CCGGTCCCAAATTGATCGCTAATTTCTCGAGAAATTAGCGATCAATTTGGGATTTTTG |
| Cxcl5 | chemokine (C-X-C motif) ligand 5 | TRCN0000054601 | NM_009141.1-312s1c1 | CCGGGAAGTCATAGCTAAACTGAAACTCGAGTTTCAGTTTAGCTATGACTTCTTTTTG |
| Cxcl9 | chemokine (C-X-C motif) ligand 9 | TRCN0000067293 | NM_008599.1-1075s1c1 | CCGGCCATCTTCAGAGCTTATTCTACTCGAGTAGAATAAGCTCTGAAGATGGTTTTTG |
| Cxcl9 | chemokine (C-X-C motif) ligand 9 | TRCN0000067294 | NM_008599.1-284s1c1 | CCGGGCTACACTGAAGAACGGAGATCTCGAGATCTCCGTTCTTCAGTGTAGCTTTTTG |
| Cxcl9 | chemokine (C-X-C motif) ligand 9 | TRCN0000067295 | NM_008599.1-450s1c1 | CCGGGTCGTCGTTCAAGGAAGACTACTCGAGTAGTCTTCCTTGAACGACGACTTTTTG |
| Ebi3 | Epstein-Barr virus induced gene 3 | TRCN0000089278 | NM_015766.2-1028s1c1 | CCGGGCTCCGAAGCACTGGATAATTCTCGAGAATTATCCAGTGCTTCGGAGCTTTTTG |
| Ebi3 | Epstein-Barr virus induced gene 3 | TRCN0000089279 | NM_015766.2-846s1c1 | CCGGGCTCAGGACCTCACAGATTATCTCGAGATAATCTGTGAGGTCCTGAGCTTTTTG |
| Ebi3 | Epstein-Barr virus induced gene 3 | TRCN0000089280 | NM_015766.2-413s1c1 | CCGGCACGTCCTTCATTGCCACTTACTCGAGTAAGTGGCAATGAAGGACGTGTTTTTG |
| Fasl | Fas ligand (TNF superfamily, member 6) | TRCN0000066638 | NM_010177.2-1604s1c1 | CCGGGCACAAATCATTCTCTACATACTCGAGTATGTAGAGAATGATTTGTGCTTTTTG |
| Fasl | Fas ligand (TNF superfamily, member 6) | TRCN0000066639 | NM_010177.2-557s1c1 | CCGGCCAACCAAAGCCTTAAAGTATCTCGAGATACTTTAAGGCTTTGGTTGGTTTTTG |
| Fasl | Fas ligand (TNF superfamily, member 6) | TRCN0000066640 | NM_010177.2-771s1c1 | CCGGCTTCGTGTATTCCAAAGTATACTCGAGTATACTTTGGAATACACGAAGTTTTTG |
| Flt3l | FMS-like tyrosine kinase 3 ligand | TRCN0000025060 | NM_013520.2-411s1c1 | CCGGCCTGCTTAAAGATTACCCAGTCTCGAGACTGGGTAATCTTTAAGCAGGTTTTT |
| Flt3l | FMS-like tyrosine kinase 3 ligand | TRCN0000025061 | NM_013520.2-558s1c1 | CCGGCGTCAACACCGAGATACATTTCTCGAGAAATGTATCTCGGTGTTGACGTTTTT |
| Flt3l | FMS-like tyrosine kinase 3 ligand | TRCN0000025059 | NM_013520.2-427s1c1 | CCGGCCAGTCACTGTGGCCGTCAATCTCGAGATTGACGGCCACAGTGACTGGTTTTT |
| Gdf1 | growth differentiation factor 1 | TRCN0000077058 | NM_008107.2-823s1c1 | CCGGGTCGTCTTTGACCTGTCGAATCTCGAGATTCGACAGGTCAAAGACGACTTTTTG |
| Gdf1 | growth differentiation factor 1 | TRCN0000077059 | NM_008107.2-1459s1c1 | CCGGTCCGTGCTCTTCTTCGACAATCTCGAGATTGTCGAAGAAGAGCACGGATTTTTG |
| Gdf1 | growth differentiation factor 1 | TRCN0000077060 | NM_008107.2-1494s1c1 | CCGGCCTGCGACACTACGAAGACATCTCGAGATGTCTTCGTAGTGTCGCAGGTTTTTG |
| Gdf10 | growth differentiation factor 10 | TRCN0000067928 | NM_145741.2-1636s1c1 | CCGGCCAGACAAGATGAACTCCCTTCTCGAGAAGGGAGTTCATCTTGTCTGGTTTTTG |
| Gdf10 | growth differentiation factor 10 | TRCN0000067929 | NM_145741.2-1083s1c1 | CCGGGCTACAGAGATACGACCCATTCTCGAGAATGGGTCGTATCTCTGTAGCTTTTTG |
| Gdf10 | growth differentiation factor 10 | TRCN0000067930 | NM_145741.2-633s1c1 | CCGGGCCTGTGTATTTCTTCAACTTCTCGAGAAGTTGAAGAAATACACAGGCTTTTTG |
| Gdf11 | growth differentiation factor 11 | TRCN0000067978 | XM_125935.4-1439s1c1 | CCGGGCAAAGGAACAGAGAGGCAAACTCGAGTTTGCCTCTCTGTTCCTTTGCTTTTTG |
| Gdf11 | growth differentiation factor 11 | TRCN0000067979 | XM_125935.4-757s1c1 | CCGGCGAGTCCTAGAGAACACGAAACTCGAGTTTCGTGTTCTCTAGGACTCGTTTTTG |
| Gdf11 | growth differentiation factor 11 | TRCN0000067980 | XM_125935.4-477s1c1 | CCGGCCTGCAGATCTTACGACTGAACTCGAGTTCAGTCGTAAGATCTGCAGGTTTTTG |
| Gdf15 | growth differentiation factor 15 | TRCN0000055183 | NM_011819.1-1127s1c1 | CCGGGCAGGCAACTCTTGAAGACTTCTCGAGAAGTCTTCAAGAGTTGCCTGCTTTTTG |
| Gdf15 | growth differentiation factor 15 | TRCN0000055184 | NM_011819.1-603s1c1 | CCGGGCCCTGGCAATGCCTGAACAACTCGAGTTGTTCAGGCATTGCCAGGGCTTTTTG |
| Gdf15 | growth differentiation factor 15 | TRCN0000055185 | NM_011819.1-536s1c1 | CCGGTCAACTGAGGTTCCTGCTGTTCTCGAGAACAGCAGGAACCTCAGTTGATTTTTG |
| Gdf2 | growth differentiation factor 2 | TRCN0000068023 | NM_019506.2-1977s1c1 | CCGGCGTGTATTGATGGAGTCACTACTCGAGTAGTGACTCCATCAATACACGTTTTTG |
| Gdf2 | growth differentiation factor 2 | TRCN0000068024 | NM_019506.2-1151s1c1 | CCGGCCCAAGGAATATGACGCCTATCTCGAGATAGGCGTCATATTCCTTGGGTTTTTG |
| Gdf2 | growth differentiation factor 2 | TRCN0000068025 | NM_019506.2-367s1c1 | CCGGCCAGTACATGATCGACTTGTACTCGAGTACAAGTCGATCATGTACTGGTTTTTG |
| Gdf3 | growth differentiation factor 3 | TRCN0000068073 | NM_008108.1-1019s1c1 | CCGGCCCTTCTCAATGACCACGTATCTCGAGATACGTGGTCATTGAGAAGGGTTTTTG |
| Gdf3 | growth differentiation factor 3 | TRCN0000068074 | NM_008108.1-325s1c1 | CCGGGCAGGACTTATGCTACGTGAACTCGAGTTCACGTAGCATAAGTCCTGCTTTTTG |
| Gdf3 | growth differentiation factor 3 | TRCN0000068075 | NM_008108.1-703s1c1 | CCGGCCGACTGAAGAATTTGGACTTCTCGAGAAGTCCAAATTCTTCAGTCGGTTTTTG |
| Gdf5 | growth differentiation factor 5 | TRCN0000068118 | NM_008109.1-2028s1c1 | CCGGGCTCAGGAAAGGTGTTCTTAACTCGAGTTAAGAACACCTTTCCTGAGCTTTTTG |
| Gdf5 | growth differentiation factor 5 | TRCN0000068119 | NM_008109.1-1747s1c1 | CCGGGCCAACAACGTGGTGTATAAACTCGAGTTTATACACCACGTTGTTGGCTTTTTG |
| Gdf5 | growth differentiation factor 5 | TRCN0000068120 | NM_008109.1-969s1c1 | CCGGCGTGTTTGACATCAGTGCCTTCTCGAGAAGGCACTGATGTCAAACACGTTTTTG |
| Gdf6 | growth differentiation factor 6 | TRCN0000088998 | NM_013526.1-3103s1c1 | CCGGGCCAGGAGAAATAACTTAAATCTCGAGATTTAAGTTATTTCTCCTGGCTTTTTG |
| Gdf6 | growth differentiation factor 6 | TRCN0000088999 | NM_013526.1-409s1c1 | CCGGGCTGTCAATCTACAAGACTTACTCGAGTAAGTCTTGTAGATTGACAGCTTTTTG |
| Gdf6 | growth differentiation factor 6 | TRCN0000089000 | NM_013526.1-545s1c1 | CCGGCGGAGACAGAAGTATTTGTTTCTCGAGAAACAAATACTTCTGTCTCCGTTTTTG |
| Gdf7 | growth differentiation factor 7 | TRCN0000068153 | NM_013527.1-1137s1c1 | CCGGGCCATTAGACTACGAGGCATACTCGAGTATGCCTCGTAGTCTAATGGCTTTTTG |
| Gdf7 | growth differentiation factor 7 | TRCN0000068154 | NM_013527.1-276s1c1 | CCGGCCACTTCATGATGTCGCTTTACTCGAGTAAAGCGACATCATGAAGTGGTTTTTG |
| Gdf7 | growth differentiation factor 7 | TRCN0000068155 | NM_013527.1-806s1c1 | CCGGCGCAAAGGAAAGAGAGTCTGTCTCGAGACAGACTCTCTTTCCTTTGCGTTTTTG |
| Gdf9 | growth differentiation factor 9 | TRCN0000068223 | NM_008110.1-1557s1c1 | CCGGCCAAGTGAAATGTAACTCATTCTCGAGAATGAGTTACATTTCACTTGGTTTTTG |
| Gdf9 | growth differentiation factor 9 | TRCN0000068224 | NM_008110.1-473s1c1 | CCGGGCCATGGAACACTTGCTCAAACTCGAGTTTGAGCAAGTGTTCCATGGCTTTTTG |
| Gdf9 | growth differentiation factor 9 | TRCN0000068225 | NM_008110.1-979s1c1 | CCGGGCCACTTCTTACAGCATCCTTCTCGAGAAGGATGCTGTAAGAAGTGGCTTTTTG |
| Gpi1 | glucose phosphate isomerase 1 | TRCN0000111855 | NM_008155.1-1759s1c1 | CCGGCCGTGTCCCTTCTCACCATATCTCGAGATATGGTGAGAAGGGACACGGTTTTTG |
| Gpi1 | glucose phosphate isomerase 1 | TRCN0000111856 | NM_008155.1-653s1c1 | CCGGCCTGAGACTTCCCTCTTTATACTCGAGTATAAAGAGGGAAGTCTCAGGTTTTTG |
| Grem1 | gremlin 1 | TRCN0000098300 | NM_011824.3-771s1c1 | CCGGCCCGATTCCTACTTGGCTTAACTCGAGTTAAGCCAAGTAGGAATCGGGTTTTTG |
| Grem1 | gremlin 1 | TRCN0000098301 | NM_011824.3-413s1c1 | CCGGCGCAAGTATCTGAAGCGAGATCTCGAGATCTCGCTTCAGATACTTGCGTTTTTG |
| Grem1 | gremlin 1 | TRCN0000098302 | NM_011824.3-685s1c1 | CCGGCCGTTGCATATCCATCGACTTCTCGAGAAGTCGATGGATATGCAACGGTTTTTG |
| Grem2 | gremlin 2 homolog, cysteine knot superfamily (Xenopus laevis) | TRCN0000248548 | NM_011825.1-595s21c1 | CCGGCCTTCCAATCCTGCGCTTTCTCTCGAGAGAAAGCGCAGGATTGGAAGGTTTTTG |
| Grem2 | gremlin 2 homolog, cysteine knot superfamily (Xenopus laevis) | TRCN0000248549 | NM_011825.1-290s21c1 | CCGGGCTGGTAAAGGTAGCTGAAACCTCGAGGTTTCAGCTACCTTTACCAGCTTTTTG |
| Grem2 | gremlin 2 homolog, cysteine knot superfamily (Xenopus laevis) | TRCN0000248550 | NM_011825.1-2026s21c1 | CCGGGCCAATATAGAGGGTAGTAATCTCGAGATTACTACCCTCTATATTGGCTTTTTG |
| Grn | granulin | TRCN0000089155 | NM_008175.2-190s1c1 | CCGGCCTAGAATAACGAGCCATCATCTCGAGATGATGGCTCGTTATTCTAGGTTTTTG |
| Grn | granulin | TRCN0000089153 | NM_008175.2-1920s1c1 | CCGGACTCATCCTGAGTCACCCTATCTCGAGATAGGGTGACTCAGGATGAGTTTTTTG |
| Il10 | interleukin 10 | TRCN0000066428 | NM_010548.1-989s1c1 | CCGGCCCTTTGCTATGGTGTCCTTTCTCGAGAAAGGACACCATAGCAAAGGGTTTTTG |
| Il10 | interleukin 10 | TRCN0000066429 | NM_010548.1-381s1c1 | CCGGCCCAGAAATCAAGGAGCATTTCTCGAGAAATGCTCCTTGATTTCTGGGTTTTTG |
| Il10 | interleukin 10 | TRCN0000066430 | NM_010548.1-276s1c1 | CCGGCGACTCCTTAATGCAGGACTTCTCGAGAAGTCCTGCATTAAGGAGTCGTTTTTG |
| Il11 | interleukin 11 | TRCN0000066458 | NM_008350.1-818s1c1 | CCGGCCTGTGGCTTATTTATACTTACTCGAGTAAGTATAAATAAGCCACAGGTTTTTG |
| Il11 | interleukin 11 | TRCN0000066459 | NM_008350.1-217s1c1 | CCGGGCTGCACAGATGAGAGACAAACTCGAGTTTGTCTCTCATCTGTGCAGCTTTTTG |
| Il11 | interleukin 11 | TRCN0000066460 | NM_008350.1-459s1c1 | CCGGCCGCCGTTTACAGCTCTTGATCTCGAGATCAAGAGCTGTAAACGGCGGTTTTTG |
| Il12a | interleukin 12a | TRCN0000066513 | NM_008351.1-702s1c1 | CCGGGCAGACCCTTACAGAGTGAAACTCGAGTTTCACTCTGTAAGGGTCTGCTTTTTG |
| Il12a | interleukin 12a | TRCN0000066514 | NM_008351.1-200s1c1 | CCGGCCTCCTAAACCACCTCAGTTTCTCGAGAAACTGAGGTGGTTTAGGAGGTTTTTG |
| Il12a | interleukin 12a | TRCN0000066515 | NM_008351.1-576s1c1 | CCGGGCACTTCAGAATCACAACCATCTCGAGATGGTTGTGATTCTGAAGTGCTTTTTG |
| Il12b | interleukin 12b | TRCN0000066568 | NM_008352.1-1508s1c1 | CCGGCCAGGCCCTATTATGCAAATTCTCGAGAATTTGCATAATAGGGCCTGGTTTTTG |
| Il12b | interleukin 12b | TRCN0000066569 | NM_008352.1-948s1c1 | CCGGCCATTCCTACTTCTCCCTCAACTCGAGTTGAGGGAGAAGTAGGAATGGTTTTTG |
| Il12b | interleukin 12b | TRCN0000066570 | NM_008352.1-796s1c1 | CCGGGCACGGCAGCAGAATAAATATCTCGAGATATTTATTCTGCTGCCGTGCTTTTTG |
| Il13 | interleukin 13 | TRCN0000066623 | NM_008355.1-284s1c1 | CCGGCCCTGACCAACATCTCCAATTCTCGAGAATTGGAGATGTTGGTCAGGGTTTTTG |
| Il13 | interleukin 13 | TRCN0000066624 | NM_008355.1-160s1c1 | CCGGCTGACCCTTAAGGAGCTTATTCTCGAGAATAAGCTCCTTAAGGGTCAGTTTTTG |
| Il13 | interleukin 13 | TRCN0000066625 | NM_008355.1-421s1c1 | CCGGCTGCTCAGCTACACAAAGCAACTCGAGTTGCTTTGTGTAGCTGAGCAGTTTTTG |
| Il15 | interleukin 15 | TRCN0000066668 | NM_008357.1-1056s1c1 | CCGGGCTTCCTAACAAGGAGATAATCTCGAGATTATCTCCTTGTTAGGAAGCTTTTTG |
| Il15 | interleukin 15 | TRCN0000066669 | NM_008357.1-550s1c1 | CCGGGCTGGCATTCATGTCTTCATTCTCGAGAATGAAGACATGAATGCCAGCTTTTTG |
| Il15 | interleukin 15 | TRCN0000066670 | NM_008357.1-608s1c1 | CCGGCCAACTGGATAGATGTAAGATCTCGAGATCTTACATCTATCCAGTTGGTTTTTG |
| Il16 | interleukin 16 | TRCN0000066498 | NM_010551.2-4756s1c1 | CCGGGCCTTTGAAGACTCATAACATCTCGAGATGTTATGAGTCTTCAAAGGCTTTTTG |
| Il16 | interleukin 16 | TRCN0000066499 | NM_010551.2-1430s1c1 | CCGGCCTGACTCTCAATGAAGTCTACTCGAGTAGACTTCATTGAGAGTCAGGTTTTTG |
| Il16 | interleukin 16 | TRCN0000066500 | NM_010551.2-891s1c1 | CCGGGACAGCATTTACGGCCCTATTCTCGAGAATAGGGCCGTAAATGCTGTCTTTTTG |
| Il17a | interleukin 17A | TRCN0000066719 | NM_010552.2-187s1c1 | CCGGCCTCCAGAATGTGAAGGTCAACTCGAGTTGACCTTCACATTCTGGAGGTTTTTG |
| Il17a | interleukin 17A | TRCN0000066720 | NM_010552.2-457s1c1 | CCGGCTTCACTTTCAGGGTCGAGAACTCGAGTTCTCGACCCTGAAAGTGAAGTTTTTG |
| Il17a | interleukin 17A | TRCN0000066721 | NM_010552.2-297s1c1 | CCGGACCGCAATGAAGACCCTGATACTCGAGTATCAGGGTCTTCATTGCGGTTTTTTG |
| Il17b | interleukin 17B | TRCN0000066758 | NM_019508.1-284s1c1 | CCGGCCAGCCAAGAAGAAATGTGAACTCGAGTTCACATTTCTTCTTGGCTGGTTTTTG |
| Il17b | interleukin 17B | TRCN0000066760 | NM_019508.1-227s1c1 | CCGGGAAGAGTATGAGCGGAACCTTCTCGAGAAGGTTCCGCTCATACTCTTCTTTTTG |
| Il17b | interleukin 17B | TRCN0000066761 | NM_019508.1-214s1c1 | CCGGCTACGCTCGAATGGAAGAGTACTCGAGTACTCTTCCATTCGAGCGTAGTTTTTG |
| Il17c | interleukin 17C | TRCN0000066808 | NM_145834.1-95s1c1 | CCGGGCTACTCTGCTGAGGAATTATCTCGAGATAATTCCTCAGCAGAGTAGCTTTTTG |
| Il17c | interleukin 17C | TRCN0000066809 | NM_145834.1-321s1c1 | CCGGTCGCATCGACACAGATGAGAACTCGAGTTCTCATCTGTGTCGATGCGATTTTTG |
| Il17c | interleukin 17C | TRCN0000066810 | NM_145834.1-28s1c1 | CCGGCCTACTGGGATGACCCACCAACTCGAGTTGGTGGGTCATCCCAGTAGGTTTTTG |
| Il17f | interleukin 17F | TRCN0000067414 | NM_145856.1-142s1c1 | CCGGGACATTCGAATCTTCAACCAACTCGAGTTGGTTGAAGATTCGAATGTCTTTTTG |
| Il17f | interleukin 17F | TRCN0000067415 | NM_145856.1-306s1c1 | CCGGTCAGGAAGACAGCACCATGAACTCGAGTTCATGGTGCTGTCTTCCTGATTTTTG |
| Il17f | interleukin 17F | TRCN0000067413 | NM_145856.1-170s1c1 | CCGGGCATTTCTGTCCCACGTGAATCTCGAGATTCACGTGGGACAGAAATGCTTTTTG |
| Il18 | interleukin 18 | TRCN0000054990 | NM_008360.1-455s1c1 | CCGGCCTCTCTGTGAAGGATAGTAACTCGAGTTACTATCCTTCACAGAGAGGTTTTTG |
| Il18 | interleukin 18 | TRCN0000054991 | NM_008360.1-660s1c1 | CCGGGCTTTCAAACTCATTCTGAAACTCGAGTTTCAGAATGAGTTTGAAAGCTTTTTG |
| Il18 | interleukin 18 | TRCN0000054992 | NM_008360.1-191s1c1 | CCGGCGTCAACTTCAAGGAAATGATCTCGAGATCATTTCCTTGAAGTTGACGTTTTTG |
| Il19 | interleukin 19 | TRCN0000066953 | XM_283649.2-962s1c1 | CCGGGCTGTGGTATTTATAGCAATACTCGAGTATTGCTATAAATACCACAGCTTTTTG |
| Il19 | interleukin 19 | TRCN0000066954 | XM_283649.2-616s1c1 | CCGGGCCTGGATTGACAGGAATCATCTCGAGATGATTCCTGTCAATCCAGGCTTTTTG |
| Il19 | interleukin 19 | TRCN0000066955 | XM_283649.2-270s1c1 | CCGGGCAAACTAAGGACACCTTTAACTCGAGTTAAAGGTGTCCTTAGTTTGCTTTTTG |
| Il1a | interleukin 1 alpha | TRCN0000067048 | NM_010554.3-1782s1c1 | CCGGCCAACCATCTAAAGATGGAAACTCGAGTTTCCATCTTTAGATGGTTGGTTTTTG |
| Il1a | interleukin 1 alpha | TRCN0000067049 | NM_010554.3-420s1c1 | CCGGCCAGAGTGATTTGAGATACAACTCGAGTTGTATCTCAAATCACTCTGGTTTTTG |
| Il1a | interleukin 1 alpha | TRCN0000067050 | NM_010554.3-778s1c1 | CCGGGCTGCTTATCCAGAGCTGTTTCTCGAGAAACAGCTCTGGATAAGCAGCTTTTTG |
| Il1b | interleukin 1 beta | TRCN0000067103 | NM_008361.2-1069s1c1 | CCGGGCAACCACTTACCTATTTATTCTCGAGAATAAATAGGTAAGTGGTTGCTTTTTG |
| Il1b | interleukin 1 beta | TRCN0000067104 | NM_008361.2-602s1c1 | CCGGCCTCAAAGGAAAGAATCTATACTCGAGTATAGATTCTTTCCTTTGAGGTTTTTG |
| Il1b | interleukin 1 beta | TRCN0000067105 | NM_008361.2-506s1c1 | CCGGCCACCTCAATGGACAGAATATCTCGAGATATTCTGTCCATTGAGGTGGTTTTTG |
| Il1f10 | interleukin 1 family, member 10 | TRCN0000067163 | NM_153077.1-290s1c1 | CCGGGACCTATACAAGGGAGGTGAACTCGAGTTCACCTCCCTTGTATAGGTCTTTTTG |
| Il1f10 | interleukin 1 family, member 10 | TRCN0000067164 | NM_153077.1-117s1c1 | CCGGGAGACCCTGATTCAGACAATTCTCGAGAATTGTCTGAATCAGGGTCTCTTTTTG |
| Il1f10 | interleukin 1 family, member 10 | TRCN0000067165 | NM_153077.1-143s1c1 | CCGGCCAGAGAAGGTCTGTATCCTTCTCGAGAAGGATACAGACCTTCTCTGGTTTTTG |
| Il1f5 | interleukin 1 family, member 5 (delta) | TRCN0000067213 | NM_019451.1-205s1c1 | CCGGGCACGCAGAGAAGGTCATTAACTCGAGTTAATGACCTTCTCTGCGTGCTTTTTG |
| Il1f5 | interleukin 1 family, member 5 (delta) | TRCN0000067214 | NM_019451.1-545s1c1 | CCGGCCCATCACAGACTTCTACTTTCTCGAGAAAGTAGAAGTCTGTGATGGGTTTTTG |
| Il1f5 | interleukin 1 family, member 5 (delta) | TRCN0000067215 | NM_019451.1-313s1c1 | CCGGCCTATCTTGTGGGACAGAGAACTCGAGTTCTCTGTCCCACAAGATAGGTTTTTG |
| Il1f6 | interleukin 1 family, member 6 | TRCN0000067263 | NM_019450.2-576s1c1 | CCGGCCACTCATTCTGACCCAAGAACTCGAGTTCTTGGGTCAGAATGAGTGGTTTTTG |
| Il1f6 | interleukin 1 family, member 6 | TRCN0000067264 | NM_019450.2-623s1c1 | CCGGCGAGATGATTGTGGTACATTACTCGAGTAATGTACCACAATCATCTCGTTTTTG |
| Il1f6 | interleukin 1 family, member 6 | TRCN0000067265 | NM_019450.2-278s1c1 | CCGGGCAAACAGTTCCAGTCACTATCTCGAGATAGTGACTGGAACTGTTTGCTTTTTG |
| Il1f8 | interleukin 1 family, member 8 | TRCN0000067298 | XM_130058.1-272s1c1 | CCGGCCTGTCATTCTTAGCTTGATACTCGAGTATCAAGCTAAGAATGACAGGTTTTTG |
| Il1f8 | interleukin 1 family, member 8 | TRCN0000067299 | XM_130058.1-525s1c1 | CCGGGCTGGTTTATAGCCACCTCTTCTCGAGAAGAGGTGGCTATAAACCAGCTTTTTG |
| Il1f8 | interleukin 1 family, member 8 | TRCN0000067300 | XM_130058.1-343s1c1 | CCGGCCTGGGAATCAAGAACAGAAACTCGAGTTTCTGTTCTTGATTCCCAGGTTTTTG |
| Il1f9 | interleukin 1 family, member 9 | TRCN0000067353 | NM_153511.1-188s1c1 | CCGGGCAGGTGTGGATCTTTCGTAACTCGAGTTACGAAAGATCCACACCTGCTTTTTG |
| Il1f9 | interleukin 1 family, member 9 | TRCN0000067354 | NM_153511.1-475s1c1 | CCGGCCACCTTTGAATCAGTGGCTTCTCGAGAAGCCACTGATTCAAAGGTGGTTTTTG |
| Il1f9 | interleukin 1 family, member 9 | TRCN0000067355 | NM_153511.1-332s1c1 | CCGGGAATCCAGATAAATGCCTGTTCTCGAGAACAGGCATTTATCTGGATTCTTTTTG |
| Il2 | interleukin 2 | TRCN0000067198 | NM_008366.2-396s1c1 | CCGGGCTGAGAATTTCATCAGCAATCTCGAGATTGCTGATGAAATTCTCAGCTTTTTG |
| Il2 | interleukin 2 | TRCN0000067199 | NM_008366.2-536s1c1 | CCGGCATCTCAACAAGCCCTCAATACTCGAGTATTGAGGGCTTGTTGAGATGTTTTTG |
| Il2 | interleukin 2 | TRCN0000067200 | NM_008366.2-221s1c1 | CCGGCCTGAGCAGGATGGAGAATTACTCGAGTAATTCTCCATCCTGCTCAGGTTTTTG |
| Il20 | interleukin 20 | TRCN0000067338 | NM_021380.1-623s1c1 | CCGGAGAACACTCCTGTCCAAGAATCTCGAGATTCTTGGACAGGAGTGTTCTTTTTTG |
| Il20 | interleukin 20 | TRCN0000067339 | NM_021380.1-320s1c1 | CCGGCGTCATCTAGTGAGATTCTATCTCGAGATAGAATCTCACTAGATGACGTTTTTG |
| Il20 | interleukin 20 | TRCN0000067340 | NM_021380.1-179s1c1 | CCGGGCAAACCTACAGGCAATACAACTCGAGTTGTATTGCCTGTAGGTTTGCTTTTTG |
| Il21 | interleukin 21 | TRCN0000067398 | NM_021782.1-676s1c1 | CCGGCCCATTAACTAAGCAGACATTCTCGAGAATGTCTGCTTAGTTAATGGGTTTTTG |
| Il21 | interleukin 21 | TRCN0000067399 | NM_021782.1-296s1c1 | CCGGCCCTGGAAACAATAAGACATTCTCGAGAATGTCTTATTGTTTCCAGGGTTTTTG |
| Il21 | interleukin 21 | TRCN0000067400 | NM_021782.1-287s1c1 | CCGGGCCATCAAACCCTGGAAACAACTCGAGTTGTTTCCAGGGTTTGATGGCTTTTTG |
| Il22 | interleukin 22 | TRCN0000067013 | NM_016971.1-525s1c1 | CCGGAGTGGAGAGATCAAGGCGATTCTCGAGAATCGCCTTGATCTCTCCACTTTTTTG |
| Il22 | interleukin 22 | TRCN0000067014 | NM_016971.1-560s1c1 | CCGGGCTGTTTATGTCTCTGAGAAACTCGAGTTTCTCAGAGACATAAACAGCTTTTTG |
| Il22 | interleukin 22 | TRCN0000067015 | NM_016971.1-464s1c1 | CCGGCGACCAGAACATCCAGAAGAACTCGAGTTCTTCTGGATGTTCTGGTCGTTTTTG |
| Il23a | interleukin 23, alpha subunit p19 | TRCN0000067120 | NM_031252.1-593s1c1 | CCGGCTTCTCCGTTCCAAGATCCTTCTCGAGAAGGATCTTGGAACGGAGAAGTTTTTG |
| Il23a | interleukin 23, alpha subunit p19 | TRCN0000067121 | NM_031252.1-221s1c1 | CCGGTCTCGGAATCTCTGCATGCTACTCGAGTAGCATGCAGAGATTCCGAGATTTTTG |
| Il24 | interleukin 24 | TRCN0000065943 | NM_053095.1-699s1c1 | CCGGCCGCAGAGCATTCAAACAGTTCTCGAGAACTGTTTGAATGCTCTGCGGTTTTTG |
| Il24 | interleukin 24 | TRCN0000065944 | NM_053095.1-286s1c1 | CCGGCTGAGCCTAATCCTTCTTCTTCTCGAGAAGAAGAAGGATTAGGCTCAGTTTTTG |
| Il24 | interleukin 24 | TRCN0000065945 | NM_053095.1-730s1c1 | CCGGGTCGCTTTGGTGAAAGCCTTTCTCGAGAAAGGCTTTCACCAAAGCGACTTTTTG |
| Il25 | interleukin 25 | TRCN0000066968 | NM_080729.1-253s1c1 | CCGGGAGTTGGACAGGGACTTGAATCTCGAGATTCAAGTCCCTGTCCAACTCTTTTTG |
| Il25 | interleukin 25 | TRCN0000066969 | NM_080729.1-378s1c1 | CCGGCCACAACCAGACGGTCTTCTACTCGAGTAGAAGACCGTCTGGTTGTGGTTTTTG |
| Il25 | interleukin 25 | TRCN0000066970 | NM_080729.1-366s1c1 | CCGGCGTCCCACTTTACCACAACCACTCGAGTGGTTGTGGTAAAGTGGGACGTTTTTG |
| Il27 | interleukin 27 | TRCN0000067088 | NM_145636.1-239s1c1 | CCGGGATACCATCTTCCCAATGTTTCTCGAGAAACATTGGGAAGATGGTATCTTTTTG |
| Il27 | interleukin 27 | TRCN0000067089 | NM_145636.1-459s1c1 | CCGGTGCAGGATTCAAATGTTCAAACTCGAGTTTGAACATTTGAATCCTGCATTTTTG |
| Il27 | interleukin 27 | TRCN0000067090 | NM_145636.1-56s1c1 | CCGGTGCTTCTGGTACAAGCTGGTTCTCGAGAACCAGCTTGTACCAGAAGCATTTTTG |
| Il3 | interleukin 3 | TRCN0000077023 | NM_010556.2-247s1c1 | CCGGCGGAGAGTAAACCTGTCCAAACTCGAGTTTGGACAGGTTTACTCTCCGTTTTTG |
| Il3 | interleukin 3 | TRCN0000077024 | NM_010556.2-129s1c1 | CCGGCCACCGTTTAACCAGAACGTTCTCGAGAACGTTCTGGTTAAACGGTGGTTTTTG |
| Il3 | interleukin 3 | TRCN0000077025 | NM_010556.2-158s1c1 | CCGGGCTCTATTGTCAAGGAGATTACTCGAGTAATCTCCTTGACAATAGAGCTTTTTG |
| Il34 | interleukin 34 | TRCN0000247082 | NM_029646.2-708s21c1 | CCGGACCGGCTTCAGTACATGAAACCTCGAGGTTTCATGTACTGAAGCCGGTTTTTTG |
| Il34 | interleukin 34 | TRCN0000247083 | NM_029646.2-1293s21c1 | CCGGATGCAATGTACAGCTACAAATCTCGAGATTTGTAGCTGTACATTGCATTTTTTG |
| Il34 | interleukin 34 | TRCN0000247084 | NM_029646.2-640s21c1 | CCGGATGGACTCTGACCCAAGATAACTCGAGTTATCTTGGGTCAGAGTCCATTTTTTG |
| Il5 | interleukin 5 | TRCN0000067503 | NM_010558.1-679s1c1 | CCGGGCCAAGGATAACCTTGAATTTCTCGAGAAATTCAAGGTTATCCTTGGCTTTTTG |
| Il5 | interleukin 5 | TRCN0000067504 | NM_010558.1-324s1c1 | CCGGAGAAATACATTGACCGCCAAACTCGAGTTTGGCGGTCAATGTATTTCTTTTTTG |
| Il5 | interleukin 5 | TRCN0000067505 | NM_010558.1-223s1c1 | CCGGGCTATGCATTGGAGAAATCTTCTCGAGAAGATTTCTCCAATGCATAGCTTTTTG |
| Il7 | interleukin 7 | TRCN0000067603 | NM_008371.2-498s1c1 | CCGGGCTCGCAAGTTGAAGCAATTTCTCGAGAAATTGCTTCAACTTGCGAGCTTTTTG |
| Il7 | interleukin 7 | TRCN0000067604 | NM_008371.2-642s1c1 | CCGGGCATGTTTCCTAAAGAGACTACTCGAGTAGTCTCTTTAGGAAACATGCTTTTTG |
| Il9 | interleukin 9 | TRCN0000067643 | NM_008373.1-188s1c1 | CCGGCCGTCCCAACTGATGATTGTACTCGAGTACAATCATCAGTTGGGACGGTTTTTG |
| Il9 | interleukin 9 | TRCN0000067644 | NM_008373.1-367s1c1 | CCGGGCAGGCAACACACTGTCATTTCTCGAGAAATGACAGTGTGTTGCCTGCTTTTTG |
| Il9 | interleukin 9 | TRCN0000067645 | NM_008373.1-100s1c1 | CCGGGACACCAATTACCTTATTGAACTCGAGTTCAATAAGGTAATTGGTGTCTTTTTG |
| Iltifb | interleukin 10-related T cell-derived inducible factor beta | TRCN0000067058 | NM_054079.1-488s1c1 | CCGGAGGCTGAAGGAGACAGTGAAACTCGAGTTTCACTGTCTCCTTCAGCCTTTTTTG |
| Iltifb | interleukin 10-related T cell-derived inducible factor beta | TRCN0000067059 | NM_054079.1-202s1c1 | CCGGCATCGTCAACCGCACCTTTATCTCGAGATAAAGGTGCGGTTGACGATGTTTTTG |
| Iltifb | interleukin 10-related T cell-derived inducible factor beta | TRCN0000067060 | NM_054079.1-298s1c1 | CCGGCAGTGCTAAGGATCAGTGCTACTCGAGTAGCACTGATCCTTAGCACTGTTTTTG |
| Kitl | kit ligand | TRCN0000067871 | NM_013598.1-405s1c1 | CCGGCTACGAGATATGGTAATACAACTCGAGTTGTATTACCATATCTCGTAGTTTTTG |
| Kitl | kit ligand | TRCN0000067872 | NM_013598.1-897s1c1 | CCGGGCCTTATACTGGAAGAAGAAACTCGAGTTTCTTCTTCCAGTATAAGGCTTTTTG |
| Lefty1 | left right determination factor 1 | TRCN0000067908 | NM_010094.2-238s1c1 | CCGGGCCCTGCTACAACACAGCCATCTCGAGATGGCTGTGTTGTAGCAGGGCTTTTTG |
| Lefty1 | left right determination factor 1 | TRCN0000067911 | NM_010094.2-80s1c1 | CCGGTCTGGGCACTGTCGCTGGTTACTCGAGTAACCAGCGACAGTGCCCAGATTTTTG |
| Lefty1 | left right determination factor 1 | TRCN0000067909 | NM_010094.2-486s1c1 | CCGGCACCATTGAATGGCTGCGCTTCTCGAGAAGCGCAGCCATTCAATGGTGTTTTTG |
| Lefty2 | Left-right determination factor 2 | TRCN0000066113 | NM_177099.3-500s1c1 | CCGGCACCATTGAATGGCTGAGAGTCTCGAGACTCTCAGCCATTCAATGGTGTTTTTG |
| Lefty2 | Left-right determination factor 2 | TRCN0000066114 | NM_177099.3-1043s1c1 | CCGGGCCCATGATTGTCAGTGTGAACTCGAGTTCACACTGACAATCATGGGCTTTTTG |
| Lefty2 | Left-right determination factor 2 | TRCN0000066115 | NM_177099.3-138s1c1 | CCGGGAACAGGTCCTGAGCAGTCTACTCGAGTAGACTGCTCAGGACCTGTTCTTTTTG |
| Lif | leukemia inhibitory factor | TRCN0000067993 | NM_008501.1-347s1c1 | CCGGGCGCCAATGCTCTCTTCATTTCTCGAGAAATGAAGAGAGCATTGGCGCTTTTTG |
| Lif | leukemia inhibitory factor | TRCN0000067996 | NM_008501.1-429s1c1 | CCGGCATGACAGACTTCCCATCTTTCTCGAGAAAGATGGGAAGTCTGTCATGTTTTTG |
| Lta | lymphotoxin A | TRCN0000068038 | NM_010735.1-423s1c1 | CCGGTGGCTTCTCTTTGAGCAACAACTCGAGTTGTTGCTCAAAGAGAAGCCATTTTTG |
| Lta | lymphotoxin A | TRCN0000068039 | NM_010735.1-547s1c1 | CCGGGCACACGAGGTCCAGCTCTTTCTCGAGAAAGAGCTGGACCTCGTGTGCTTTTTG |
| Lta | lymphotoxin A | TRCN0000068040 | NM_010735.1-613s1c1 | CCGGTCTGTGTATCCGGGACTTCAACTCGAGTTGAAGTCCCGGATACACAGATTTTTG |
| Ltb | lymphotoxin B | TRCN0000068090 | NM_008518.1-834s1c1 | CCGGCGAGAGGGTCTACGTTAACATCTCGAGATGTTAACGTAGACCCTCTCGTTTTTG |
| Ltb | lymphotoxin B | TRCN0000068091 | NM_008518.1-518s1c1 | CCGGGCCAAGAAGAAGCGTTTCTGACTCGAGTCAGAAACGCTTCTTCTTGGCTTTTTG |
| Mif | macrophage migration inhibitory factor | TRCN0000067343 | NM_010798.1-449s1c1 | CCGGGCACCGCTGTTCTTTGAGCCTCTCGAGAGGCTCAAAGAACAGCGGTGCTTTTTG |
| Mif | macrophage migration inhibitory factor | TRCN0000067344 | NM_010798.1-360s1c1 | CCGGCCGGGTCTACATCAACTATTACTCGAGTAATAGTTGATGTAGACCCGGTTTTTG |
| Mif | macrophage migration inhibitory factor | TRCN0000067345 | NM_010798.1-294s1c1 | CCGGCCAGAACCGCAACTACAGTAACTCGAGTTACTGTAGTTGCGGTTCTGGTTTTTG |
| Nodal | nodal | TRCN0000068168 | NM_013611.3-823s1c1 | CCGGCCCAAGCAGTACAATGCCTATCTCGAGATAGGCATTGTACTGCTTGGGTTTTTG |
| Nodal | nodal | TRCN0000068169 | NM_013611.3-219s1c1 | CCGGCTGGACTTTCACGTTTGACTTCTCGAGAAGTCAAACGTGAAAGTCCAGTTTTTG |
| Nodal | nodal | TRCN0000068170 | NM_013611.3-778s1c1 | CCGGCAGGTGGACTTCAACCTGATTCTCGAGAATCAGGTTGAAGTCCACCTGTTTTTG |
| Osm | oncostatin M | TRCN0000065593 | XM_137493.3-465s1c1 | CCGGCGGCACAATATCCTCGGCATACTCGAGTATGCCGAGGATATTGTGCCGTTTTTG |
| Osm | oncostatin M | TRCN0000065594 | XM_137493.3-580s1c1 | CCGGCAACACCAGATGTCTTTAATACTCGAGTATTAAAGACATCTGGTGTTGTTTTTG |
| Osm | oncostatin M | TRCN0000065595 | XM_137493.3-234s1c1 | CCGGGAATCACTCTTGGAGCCCTATCTCGAGATAGGGCTCCAAGAGTGATTCTTTTTG |
| Pglyrp1 | peptidoglycan recognition protein 1 | TRCN0000065598 | NM_009402.1-435s1c1 | CCGGGCTGCCCTAAATCTTCTGGAACTCGAGTTCCAGAAGATTTAGGGCAGCTTTTTG |
| Pglyrp1 | peptidoglycan recognition protein 1 | TRCN0000065599 | NM_009402.1-355s1c1 | CCGGCCATCTGGAATCCCATGTCTACTCGAGTAGACATGGGATTCCAGATGGTTTTTG |
| Ppbp | pro-platelet basic protein | TRCN0000301445 | NM_023785.2-130s21c1 | CCGGCCCACTTCATAACCTCCAGATCTCGAGATCTGGAGGTTATGAAGTGGGTTTTTG |
| Ppbp | pro-platelet basic protein | TRCN0000301446 | NM_023785.2-636s21c1 | CCGGCCTGCAATTTATGGTCTATTTCTCGAGAAATAGACCATAAATTGCAGGTTTTTG |
| Ppbp | pro-platelet basic protein | TRCN0000301448 | NM_023785.2-388s21c1 | CCGGCGTCAAGAGAATCGTCATGAACTCGAGTTCATGACGATTCTCTTGACGTTTTTG |
| Scg2 | secretogranin II | TRCN0000201714 | NM_009129.1-328s1c1 | CCGGGACGAGTGGATGCGGATAATACTCGAGTATTATCCGCATCCACTCGTCTTTTTTG |
| Scg2 | secretogranin II | TRCN0000217809 | NM_009129.2-2244s1c1 | CCGGCTATGATAGTGTTGGCTAATGCTCGAGCATTAGCCAACACTATCATAGTTTTTTG |
| Scg2 | secretogranin II | TRCN0000219456 | NM_009129.2-1670s1c1 | CCGGCTTAGCCAGGATGCTAGTTAACTCGAGTTAACTAGCATCCTGGCTAAGTTTTTG |
| Scgb3a1 | secretoglobin, family 3A, member 1 | TRCN0000190171 | NM_170727.1-305s1c1 | CCGGCTAGCCTACCATTAAGCCACTCTCGAGAGTGGCTTAATGGTAGGCTAGTTTTTTG |
| Scgb3a1 | secretoglobin, family 3A, member 1 | TRCN0000192491 | NM_054037.1-102s1c1 | CCGGCTCTGGTGTTGCTTTCTTCATCTCGAGATGAAGAAAGCAACACCAGAGTTTTTTG |
| Scgb3a1 | secretoglobin, family 3A, member 1 | TRCN0000202129 | NM_170727.1-364s1c1 | CCGGCCATTGGATCCTCTCATAGAGCTCGAGCTCTATGAGAGGATCCAATGGTTTTTTG |
| Slurp1 | secreted Ly6/Plaur domain containing 1 | TRCN0000190210 | NM_020519.1-174s1c1 | CCGGCCTGTAAGACTGTACTGGAGACTCGAGTCTCCAGTACAGTCTTACAGGTTTTTTG |
| Slurp1 | secreted Ly6/Plaur domain containing 1 | TRCN0000190254 | NM_020519.1-94s1c1 | CCGGCTTCCGATGCTATACCTGTGACTCGAGTCACAGGTATAGCATCGGAAGTTTTTTG |
| Slurp1 | secreted Ly6/Plaur domain containing 1 | TRCN0000192879 | NM_020519.1-125s1c1 | CCGGGCCATTAACTCATGCAAGAATCTCGAGATTCTTGCATGAGTTAATGGCTTTTTTG |
| Spp1 | secreted phosphoprotein 1 | TRCN0000009602 | NM_009263.1-678s1c1 | CCGGGAGGTCAAAGTCTAGGAGTTTCTCGAGAAACTCCTAGACTTTGACCTCTTTTT |
| Spp1 | secreted phosphoprotein 1 | TRCN0000054699 | NM_009263.1-416s1c1 | CCGGAGGATGACTTTAAGCAAGAAACTCGAGTTTCTTGCTTAAAGTCATCCTTTTTTG |
| Spp1 | secreted phosphoprotein 1 | TRCN0000009601 | NM_009263.1-1178s1c1 | CCGGGCAGGAATACTAACTGCTCATCTCGAGATGAGCAGTTAGTATTCCTGCTTTTT |
| Spp1 | secreted phosphoprotein 1 | TRCN0000009603 | NM_009263.1-1027s1c1 | CCGGCCTAAGAGTAAGGAAGATGATCTCGAGATCATCTTCCTTACTCTTAGGTTTTT |
| Spp1 | secreted phosphoprotein 1 | TRCN0000009604 | NM_009263.1-272s1c1 | CCGGTCCCGGTGAAAGTGACTGATTCTCGAGAATCAGTCACTTTCACCGGGATTTTT |
| Thpo | thrombopoietin | TRCN0000066843 | NM_009379.2-1112s1c1 | CCGGCCCAGTCCAAATCTCTGGATACTCGAGTATCCAGAGATTTGGACTGGGTTTTTG |
| Thpo | thrombopoietin | TRCN0000066846 | NM_009379.2-554s1c1 | CCGGCCCTTTGTCTATCCCTGTTCTCTCGAGAGAACAGGGATAGACAAAGGGTTTTTG |
| Tnf | tumor necrosis factor | TRCN0000066183 | NM_013693.1-640s1c1 | CCGGGCTATCTCATACCAGGAGAAACTCGAGTTTCTCCTGGTATGAGATAGCTTTTTG |
| Tnf | tumor necrosis factor | TRCN0000066184 | NM_013693.1-549s1c1 | CCGGCGATGGGTTGTACCTTGTCTACTCGAGTAGACAAGGTACAACCCATCGTTTTTG |
| Tnf | tumor necrosis factor | TRCN0000066186 | NM_013693.1-357s1c1 | CCGGCCTCCCTCTCATCAGTTCTATCTCGAGATAGAACTGATGAGAGGGAGGTTTTTG |
| Tnfsf10 | tumor necrosis factor (ligand) superfamily, member 10 | TRCN0000066234 | NM_009425.1-449s1c1 | CCGGCGGAGAAGCAACTCAGCTTTACTCGAGTAAAGCTGAGTTGCTTCTCCGTTTTTG |
| Tnfsf10 | tumor necrosis factor (ligand) superfamily, member 10 | TRCN0000066235 | NM_009425.1-649s1c1 | CCGGCGCTTCCAAGATGGTCTCAAACTCGAGTTTGAGACCATCTTGGAAGCGTTTTTG |
| Tnfsf10 | tumor necrosis factor (ligand) superfamily, member 10 | TRCN0000066236 | NM_009425.1-316s1c1 | CCGGGCTCATTGAAGAGGTGACTTTCTCGAGAAAGTCACCTCTTCAATGAGCTTTTTG |
| Tnfsf11 | tumor necrosis factor (ligand) superfamily, member 11, RANKL | TRCN0000066284 | NM_011613.2-913s1c1 | CCGGCCCAAGTTCTCATAACCTGATCTCGAGATCAGGTTATGAGAACTTGGGTTTTTG |
| Tnfsf11 | tumor necrosis factor (ligand) superfamily, member 11, RANKL | TRCN0000066285 | NM_011613.2-387s1c1 | CCGGCGCAGATGGATCCTAACAGAACTCGAGTTCTGTTAGGATCCATCTGCGTTTTTG |
| Tnfsf11 | tumor necrosis factor (ligand) superfamily, member 11, RANKL | TRCN0000066286 | NM_011613.2-760s1c1 | CCGGCATGACGTTAAGCAACGGAAACTCGAGTTTCCGTTGCTTAACGTCATGTTTTTG |
| Tnfsf11 | tumor necrosis factor (ligand) superfamily, member 11, RANKL | TRCN0000066283 | NM_011613.2-1094s1c1 | CCGGGCTTTCAAAGTTCAGGACATACTCGAGTATGTCCTGAACTTTGAAAGCTTTTTG |
| Tnfsf11 | tumor necrosis factor (ligand) superfamily, member 11, RANKL | TRCN0000066287 | NM_011613.2-877s1c1 | CCGGGCTGATGGTGTATGTCGTTAACTCGAGTTAACGACATACACCATCAGCTTTTTG |
| Tnfsf12 | tumor necrosis factor (ligand) superfamily, member 12 | TRCN0000066324 | NM_011614.1-717s1c1 | CCGGCCTAACCTACTTTGGACTCTTCTCGAGAAGAGTCCAAAGTAGGTTAGGTTTTTG |
| Tnfsf12 | tumor necrosis factor (ligand) superfamily, member 12 | TRCN0000066325 | NM_011614.1-271s1c1 | CCGGCCTCGAAGAAGTGCTCCTAAACTCGAGTTTAGGAGCACTTCTTCGAGGTTTTTG |
| Tnfsf12 | tumor necrosis factor (ligand) superfamily, member 12 | TRCN0000066326 | NM_011614.1-244s1c1 | CCGGGTACCTTTCTTGGAACAACTACTCGAGTAGTTGTTCCAAGAAAGGTACTTTTTG |
| Tnfsf13 | tumor necrosis factor (ligand) superfamily, member 13 | TRCN0000076983 | NM_023517.1-1334s1c1 | CCGGTGGCTAGACAAAGGACAAGGACTCGAGTCCTTGTCCTTTGTCTAGCCATTTTTG |
| Tnfsf13 | tumor necrosis factor (ligand) superfamily, member 13 | TRCN0000076984 | NM_023517.1-628s1c1 | CCGGCGGTTGCTCTTTGGTTGAGTTCTCGAGAACTCAACCAAAGAGCAACCGTTTTTG |
| Tnfsf13 | tumor necrosis factor (ligand) superfamily, member 13 | TRCN0000076985 | NM_023517.1-1182s1c1 | CCGGGCAGGTGTCTTTCATTTACATCTCGAGATGTAAATGAAAGACACCTGCTTTTTG |
| Tnfsf13b | tumor necrosis factor (ligand) superfamily, member 13b | TRCN0000066368 | NM_033622.1-671s1c1 | CCGGGCCATTCTCAACATGATGATACTCGAGTATCATCATGTTGAGAATGGCTTTTTG |
| Tnfsf13b | tumor necrosis factor (ligand) superfamily, member 13b | TRCN0000066369 | NM_033622.1-263s1c1 | CCGGGCTCCGAGAAAGGAGAAGATACTCGAGTATCTTCTCCTTTCTCGGAGCTTTTTG |
| Tnfsf13b | tumor necrosis factor (ligand) superfamily, member 13b | TRCN0000066370 | NM_033622.1-1079s1c1 | CCGGCTCGGGAGAATGCACAGATTTCTCGAGAAATCTGTGCATTCTCCCGAGTTTTTG |
| Tnfsf14 | tumor necrosis factor (ligand) superfamily, member 14 | TRCN0000066398 | NM_019418.1-528s1c1 | CCGGGCCCGGTTACTACTATGTGTACTCGAGTACACATAGTAGTAACCGGGCTTTTTG |
| Tnfsf14 | tumor necrosis factor (ligand) superfamily, member 14 | TRCN0000066400 | NM_019418.1-358s1c1 | CCGGGAGAAGCTGATACAAGATCAACTCGAGTTGATCTTGTATCAGCTTCTCTTTTTG |
| Tnfsf14 | tumor necrosis factor (ligand) superfamily, member 14 | TRCN0000066399 | NM_019418.1-813s1c1 | CCGGCAGGTCCTATTTCGGAGCTTTCTCGAGAAAGCTCCGAAATAGGACCTGTTTTTG |
| Tnfsf15 | tumor necrosis factor (ligand) superfamily, member 15 | TRCN0000066434 | NM_177371.1-287s1c1 | CCGGCGAGAGCACACCTGACAATTACTCGAGTAATTGTCAGGTGTGCTCTCGTTTTTG |
| Tnfsf15 | tumor necrosis factor (ligand) superfamily, member 15 | TRCN0000066435 | NM_177371.1-426s1c1 | CCGGCCCAGAGTCAGGAGACTATTTCTCGAGAAATAGTCTCCTGACTCTGGGTTTTTG |
| Tnfsf15 | tumor necrosis factor (ligand) superfamily, member 15 | TRCN0000066436 | NM_177371.1-579s1c1 | CCGGCCGCCTACTAACAGGGTCCAACTCGAGTTGGACCCTGTTAGTAGGCGGTTTTTG |
| Tnfsf4 | tumor necrosis factor (ligand) superfamily, member 4 | TRCN0000066529 | NM_009452.1-414s1c1 | CCGGGCAGAACAATTCGGTTGTCATCTCGAGATGACAACCGAATTGTTCTGCTTTTTG |
| Tnfsf4 | tumor necrosis factor (ligand) superfamily, member 4 | TRCN0000066531 | NM_009452.1-546s1c1 | CCGGCGATGGTCGAAGGATTGTCTTCTCGAGAAGACAATCCTTCGACCATCGTTTTTG |
| Tnfsf8 | tumor necrosis factor (ligand) superfamily, member 8 | TRCN0000066728 | NM_009403.2-823s1c1 | CCGGGCTGCATTACTTACAGGTCAACTCGAGTTGACCTGTAAGTAATGCAGCTTTTTG |
| Tnfsf8 | tumor necrosis factor (ligand) superfamily, member 8 | TRCN0000066729 | NM_009403.2-404s1c1 | CCGGGCGATCATTCTGGTACTGGTACTCGAGTACCAGTACCAGAATGATCGCTTTTTG |
| Tnfsf8 | tumor necrosis factor (ligand) superfamily, member 8 | TRCN0000066730 | NM_009403.2-647s1c1 | CCGGCCTGGCTTGTACTTCATCGTTCTCGAGAACGATGAAGTACAAGCCAGGTTTTTG |
| Tnfsf9 | tumor necrosis factor (ligand) superfamily, member 9 | TRCN0000077039 | NM_009404.1-175s1c1 | CCGGCCTGCGGTTAATGTTCGGGATCTCGAGATCCCGAACATTAACCGCAGGTTTTTG |
| Tnfsf9 | tumor necrosis factor (ligand) superfamily, member 9 | TRCN0000077040 | NM_009404.1-746s1c1 | CCGGAGAACAAGTTAGTGGACCGTTCTCGAGAACGGTCCACTAACTTGTTCTTTTTTG |
| Tnfsf9 | tumor necrosis factor (ligand) superfamily, member 9 | TRCN0000077041 | NM_009404.1-473s1c1 | CCGGCGTTGTGCAATACAACTCTGACTCGAGTCAGAGTTGTATTGCACAACGTTTTTG |
| Tslp | thymic stromal lymphopoietin | TRCN0000076993 | NM_021367.1-521s1c1 | CCGGCCACTGGTGTTTATTCTTTAACTCGAGTTAAAGAATAAACACCAGTGGTTTTTG |
| Tslp | thymic stromal lymphopoietin | TRCN0000076994 | NM_021367.1-418s1c1 | CCGGCCTTCATGCAATCTCCAGAATCTCGAGATTCTGGAGATTGCATGAAGGTTTTTG |
| Tslp | thymic stromal lymphopoietin | TRCN0000076995 | NM_021367.1-316s1c1 | CCGGGCTACCCTGAAACTGAGAGAACTCGAGTTCTCTCAGTTTCAGGGTAGCTTTTTG |
| Xcl1 | chemokine (C motif) ligand 1 | TRCN0000065448 | NM_008510.1-226s1c1 | CCGGGTGCTGATCCAGAAGCCAAATCTCGAGATTTGGCTTCTGGATCAGCACTTTTTG |
| Xcl1 | chemokine (C motif) ligand 1 | TRCN0000065450 | NM_008510.1-255s1c1 | CCGGGCAGCGATCAAGACTGTGGATCTCGAGATCCACAGTCTTGATCGCTGCTTTTTG |
| Xcl1 | chemokine (C motif) ligand 1 | TRCN0000065449 | NM_008510.1-101s1c1 | CCGGAGAAGAGAGTAGCTGTGTGAACTCGAGTTCACACAGCTACTCTCTTCTTTTTTG |
| Control | non-targeting hairpin |  |  | CCGGCCTAAGGTTAAGTCGCCCTCGCTCGAGCGAGGGCGACTTAACCTTAGGTTTTTG |
