# Supplementary material for: RNAi Screen Reveals Potentially Novel Roles of Cytokines in Myoblast Differentiation
Source: PLoS One. 2013 Jul 2;8(7):e68068. doi: 10.1371/journal.pone.0068068 (PMC3699544; doi:10.1371/journal.pone.0068068)
Supplement: Table S2 — Gene-specific primers for RT-PCR. (DOCX) [file pone.0068068.s002.docx]

**Table S2. Gene-specific primers for RT-PCR.**

| **Gene** | **forward primer sequence** | **reverse primer sequence** |
| --- | --- | --- |
| Ccl9 | tgcctgtcctataactcacg | tctctgaactctccgatcac |
| Ccl17 | gagtgctgcctggattactt | ggtctgcacagatgagcttg |
| Cmtm5 | gacaagaccttcctgtcttc | gtgtgatgaggaactctagc |
| Cxcl9 | atcatcttcctggagcagtg | tctccgttcttcagtgtagc |
| Cxcl10 | ctgcaactgcatccatatcg | ggattcagacatctctgctc |
| Cxcl12 | agccaacgtcaagcatctg | caggtactcttggatccac |
| Cxcl14 | ggtccaagtgtaagtgttcc | cctggacatgctcttggtg |
| Gdf3 | cgagtttcaagactctgacc | gctccttcacgtagcataag |
| Gdf15 | agaggactcgaactcagaac | tcagcaggagcagcgctc |
| IL1f9 | ttgtgacagttccacgaagc | ggtgtccattaacttccttac |
| IL18 | cctgtgttcgaggatatgac | ggagagggtagacattttac |
| Tnfα | gagcacagaaagcatgatcc | tcagtagacagaagagcgtg |
