# Supplementary material for: RNAi Screen Reveals Potentially Novel Roles of Cytokines in Myoblast Differentiation
Source: PLoS One. 2013 Jul 2;8(7):e68068. doi: 10.1371/journal.pone.0068068 (PMC3699544; doi:10.1371/journal.pone.0068068)
Supplement: Table S3 — Quantification of myotube formation for 29 candidate genes. (DOCX) [file pone.0068068.s003.docx]

**Table S3. Quantification of myotube formation for 29 candidate genes.** Differentiation (“Diff.”) index, fusion index and myotube size (average nuclei number per myotube) were calculated for the knockdown of the genes listed in Table 1. The data shown are mean$\pm$standard deviation (n=3-6). The experiments were performed in 12-well plates in several groups, with the non-targeting control shRNA included in each group for viral packaging, infection and differentiation for side-by-side comparison. Variation in control values between groups was primarily the result of varying cell densities at the induction of differentiation. Statistical analyses were performed by *t* tests to compare each data to the matching control. **P*<0.05, ***P*<0.01.

| Gene/shRNA | | Diff. index | Fusion index | Myotube size |
| --- | --- | --- | --- | --- |
| Ccl8 | Control | 0.24±0.07 | 0.20±0.07 | 11.0±5.4 |
|  | shRNA #1 | 0.13±0.05** | 0.04±0.01** | 2.5±0.2** |
|  | shRNA #2 | 0.09±0.02** | 0.04±0.01** | 2.6±0.2** |
| Cxcl9 | Control | 0.36±0.01 | 0.34±0.01 | 8.6±1.3 |
|  | shRNA #1 | 0.25±0.03* | 0.22±0.03** | 4.9±0.6* |
|  | shRNA #2 | 0.22±0.02** | 0.21±0.04* | 4.4±2.1* |
| Flt3L | Control | 0.25±0.04 | 0.22±0.03 | 6.2±0.7 |
|  | shRNA #1 | 0.15±0.02** | 0.12±0.02** | 3.8±1.2* |
|  | shRNA #2 | 0.09±0.02** | 0.07±0.02** | 2.5±0.5** |
| Tnfsf14 | Control | 0.27±0.04 | 0.24±0.04 | 13.9±1.9 |
|  | shRNA #1 | 0.25±0.04* | 0.19±0.04* | 5.0±1.5** |
|  | shRNA #2 | 0.22±0.03* | 0.12±0.03* | 2.5±0.1** |
| Gdf15 | Control | 0.29±0.03 | 0.28±0.04 | 6.0±1.4 |
|  | shRNA #1 | 0.29±0.02 | 0.17±0.05* | 3.0±0.3* |
|  | shRNA #2 | 0.26±0.05 | 0.19±0.01* | 2.6±0.5* |
| Scgb3a1 | Control | 0.25±0.04 | 0.22±0.03 | 6.2±0.7 |
|  | shRNA #1 | 0.24±0.03 | 0.15±0.03* | 3.8±0.4** |
|  | shRNA #2 | 0.24±0.03 | 0.13±0.06* | 4.3±1.3* |
| Ccl1 | Control | 0.25±0.04 | 0.22±0.03 | 6.2±0.7 |
|  | shRNA #1 | 0.34±0.02* | 0.33±0.03* | 9.2±2.8* |
|  | shRNA #2 | 0.31±0.01* | 0.29±0.03* | 10.6±4.0* |
| Cmtm5 | Control | 0.25±0.04 | 0.22±0.03 | 6.2±0.7 |
|  | shRNA #1 | 0.28±0.08* | 0.25±0.07* | 8.2±1.5* |
|  | shRNA #2 | 0.27±0.03* | 0.24±0.04* | 7.1±1.8* |

(Table S3 continued)

| Gene/shRNA | | Diff. index | Fusion index | Myotube size |
| --- | --- | --- | --- | --- |
| Cmtm6 | Control | 0.25±0.04 | 0.22±0.03 | 6.2±0.7 |
|  | shRNA #1 | 0.30±0.09* | 0.28±0.10* | 12.5±1.2** |
|  | shRNA #2 | 0.26±0.01* | 0.24±0.01* | 8.1±2.1* |
| Ctf2 | Control | 0.24±0.03 | 0.22±0.03 | 6.4±0.6 |
|  | shRNA #1 | 0.32±0.08* | 0.30±0.08* | 23.2±11.9** |
|  | shRNA #2 | 0.40±0.08** | 0.37±0.10** | 16.2±7.4** |
| Cxcl2 | Control | 0.25±0.04 | 0.22±0.03 | 6.2±0.7 |
|  | shRNA #1 | 0.43±0.05** | 0.41±0.05** | 8.3±0.2* |
|  | shRNA #2 | 0.40±0.05** | 0.39±0.05** | 21.6±10.6* |
|  | shRNA #3 | 0.33±0.06* | 0.32±0.06** | 15.6±8.1* |
| Cxcl10 | Control | 0.28±0.03 | 0.27±0.04 | 5.7±1.1 |
|  | shRNA #1 | 0.38±0.02* | 0.36±0.02* | 7.7±1.2* |
|  | shRNA #2 | 0.35±0.03* | 0.34±0.03* | 7.2±0.6* |
| Cxcl14 | Control | 0.27±0.04 | 0.24±0.04 | 13.9±1.9 |
|  | shRNA #1 | 0.64±0.01** | 0.62±0.01** | 49.7±12.1** |
|  | shRNA #2 | 0.69±0.05** | 0.66±0.06** | 45.6±18.5** |
| FasL | Control | 0.34±0.06 | 0.31±0.06 | 8.6±1.0 |
|  | shRNA #1 | 0.43±0.05** | 0.43±0.04** | 18.0±3.3** |
|  | shRNA #2 | 0.37±0.03* | 0.36±0.02* | 13.7±5.8** |
| Gdf3 | Control | 0.25±0.04 | 0.22±0.03 | 6.2±0.7 |
|  | shRNA #1 | 0.33±0.01* | 0.32±0.01* | 10.2±1.2* |
|  | shRNA #2 | 0.30±0.02* | 0.29±0.02* | 9.3±2.5* |
|  | shRNA #3 | 0.29±0.04* | 0.28±0.04* | 9.8±2.6* |
| Il5 | Control | 0.25±0.04 | 0.22±0.03 | 6.2±0.7 |
|  | shRNA #1 | 0.35±0.09* | 0.31±0.08* | 12.4±7.2* |
|  | shRNA #2 | 0.27±0.08* | 0.23±0.09* | 8.0 ±0.1* |
| Il17b | Control | 0.25±0.04 | 0.22±0.03 | 6.2±0.7 |
|  | shRNA #1 | 0.34±0.06* | 0.31±0.09* | 28.2±6.7** |
|  | shRNA #2 | 0.32±0.10* | 0.31±0.10* | 9.3±1.8* |
| Il17c | Control | 0.25±0.04 | 0.22±0.03 | 6.2±0.7 |
|  | shRNA #1 | 0.37±0.05** | 0.36±0.05** | 34.4±16.2** |
|  | shRNA #2 | 0.29±0.01* | 0.28±0.02* | 11.0±1.7** |
| Scg2 | Control | 0.25±0.04 | 0.22±0.03 | 6.2±0.7 |
|  | shRNA #1 | 0.37±0.12** | 0.36±0.11** | 22.1±8.1** |
|  | shRNA #2 | 0.32±0.06* | 0.28±0.04* | 11.3±4.5* |
| Tnfα | Control | 0.30±0.04 | 0.26±0.03 | 6.4±1.0 |
|  | shRNA #1 | 0.36±0.03* | 0.31±0.02* | 8.7±1.8* |
|  | shRNA #2 | 0.39±0.02* | 0.32±0.02* | 7.9±1.3* |

(Table S3 continued)

| Gene/shRNA | | Diff. index | Fusion index | Myotube size |
| --- | --- | --- | --- | --- |
| Tnfsf10 | Control | 0.27±0.04 | 0.24±0.04 | 13.9±1.9 |
|  | shRNA #1 | 0.43±0.05** | 0.40±0.06** | 19.0±5.1* |
|  | shRNA #2 | 0.45±0.07** | 0.43±0.08** | 40.5±13.5** |
|  | shRNA #3 | 0.51±0.06** | 0.50±0.06** | 27.2±4.0** |
| Ccl9 | Control | 0.25±0.04 | 0.22±0.03 | 6.2±0.7 |
|  | shRNA #1 | 0.29±0.04* | 0.26±0.04* | 6.3±1.1 |
|  | shRNA #2 | 0.38±0.06** | 0.34±0.06** | 6.8±0.9 |
| Ccl17 | Control | 0.25±0.04 | 0.22±0.03 | 6.2±0.7 |
|  | shRNA #1 | 0.29±0.12* | 0.27±0.13* | 7.1±4.0 |
|  | shRNA #2 | 0.34±0.06** | 0.32±0.05* | 7.6±1.9 |
| Cmtm2a | Control | 0.25±0.04 | 0.22±0.03 | 6.2±0.7 |
|  | shRNA #1 | 0.29±0.08* | 0.28±0.07* | 7.7±2.2 |
|  | shRNA #2 | 0.30±0.07* | 0.29±0.08* | 7.8±5.4 |
|  | shRNA #3 | 0.32±0.03* | 0.31±0.02* | 8.4±2.7 |
| Ebi3 | Control | 0.25±0.04 | 0.22±0.03 | 6.2±0.7 |
|  | shRNA #1 | 0.28±0.09* | 0.24±0.09* | 4.8±1.0 |
|  | shRNA #2 | 0.27±0.06* | 0.26±0.07* | 7.6±1.9 |
| Il18 | Control | 0.25±0.04 | 0.22±0.03 | 6.2±0.7 |
|  | shRNA #1 | 0.35±0.06** | 0.33±0.06** | 6.2±3.3 |
|  | shRNA #2 | 0.31±0.08* | 0.30±0.08* | 9.5±4.4 |
| Il21 | Control | 0.25±0.04 | 0.22±0.03 | 6.2±0.7 |
|  | shRNA #1 | 0.30±0.11* | 0.29±0.11* | 7.9±2.9 |
|  | shRNA #2 | 0.31±0.04* | 0.26±0.01* | 4.3±2.0 |
| Il1f9 | Control | 0.35±0.03 | 0.33±0.03 | 7.8±1.4 |
|  | shRNA #1 | 0.47±0.05* | 0.45±0.06* | 7.7±2.1 |
|  | shRNA #2 | 0.44±0.05* | 0.39±0.02* | 7.1±2.6 |
| Iltifb | Control | 0.25±0.04 | 0.22±0.03 | 6.2±0.7 |
|  | shRNA #1 | 0.47±0.06** | 0.45±0.06** | 7.9±2.5 |
|  | shRNA #2 | 0.35±0.05** | 0.32±0.06** | 5.4±2.1 |
|  | shRNA #3 | 0.35±0.07** | 0.33±0.06** | 8.6±5.7 |
